# Uncovering MYOF as a novel therapeutic target in glioblastoma: mechanistic insights and drug discovery

**DOI:** 10.1038/s41420-026-03254-0

**Published:** 2026-07-14

**Authors:** Peiqi Zhao, Zhen Chen, Jiajie Zhu, Zibin Zhang, Zhenqiu Xing, Fan Tang, Kankai Wang, Ying Zhang, Hong Chen, Qichuan Zhuge, Jianjing Yang

**Affiliations:** 1 Department of Neurosurgery, The First Affiliated Hospital of Wenzhou Medical University, Wenzhou, China; 2 Zhejiang-US Joint Laboratory for Aging and Neurological Disease Research, The First Affiliated Hospital of Wenzhou Medical University, Wenzhou, China; 3 Zhejiang Provincial Key Laboratory of Aging and Neurological Disorder Research, The First Affiliated Hospital of Wenzhou Medical University, Wenzhou, China; 4 Department of Neurosurgery, Second Affiliated Hospital, School of Medicine, Zhejiang University, Hangzhou, China; 5 Key Laboratory of Precise Treatment and Clinical Translational Research of Neurological Diseases, Hangzhou, China

**Keywords:** CNS cancer, Targeted therapies

## Abstract

Glioblastoma (GBM) is the most common type of primary malignant brain tumor, characterized by a poor prognosis, high recurrence rate, and elevated mortality. In recent years, gene-targeted therapies leveraging small molecule compounds have gained momentum as a promising avenue for GBM intervention. Myoferlin (MYOF), a type II membrane protein of the Ferlin family, has emerged as a key regulator of membrane dynamics—governing processes such as vesicular trafficking, endocytosis, and membrane repair. In this study, we explore the previously uncharted role of MYOF in GBM progression and its potential as a diagnostic and therapeutic target. Our data reveal that silencing MYOF markedly suppresses glioma growth both in vitro and in vivo. Mechanistically, MYOF knockdown disrupts the nuclear translocation of phosphorylated STAT3 (P-STAT3), a critical oncogenic signaling event. Notably, we identified Entacapone (ENT), a small molecule capable of targeting MYOF, which significantly impedes glioma development across experimental models. These findings position MYOF as a novel molecular lever in GBM pathogenesis and highlight ENT as a potential therapeutic agent that exerts anti-glioma effects by blocking MYOF-mediated P-STAT3 nuclear import.

**Figure:**
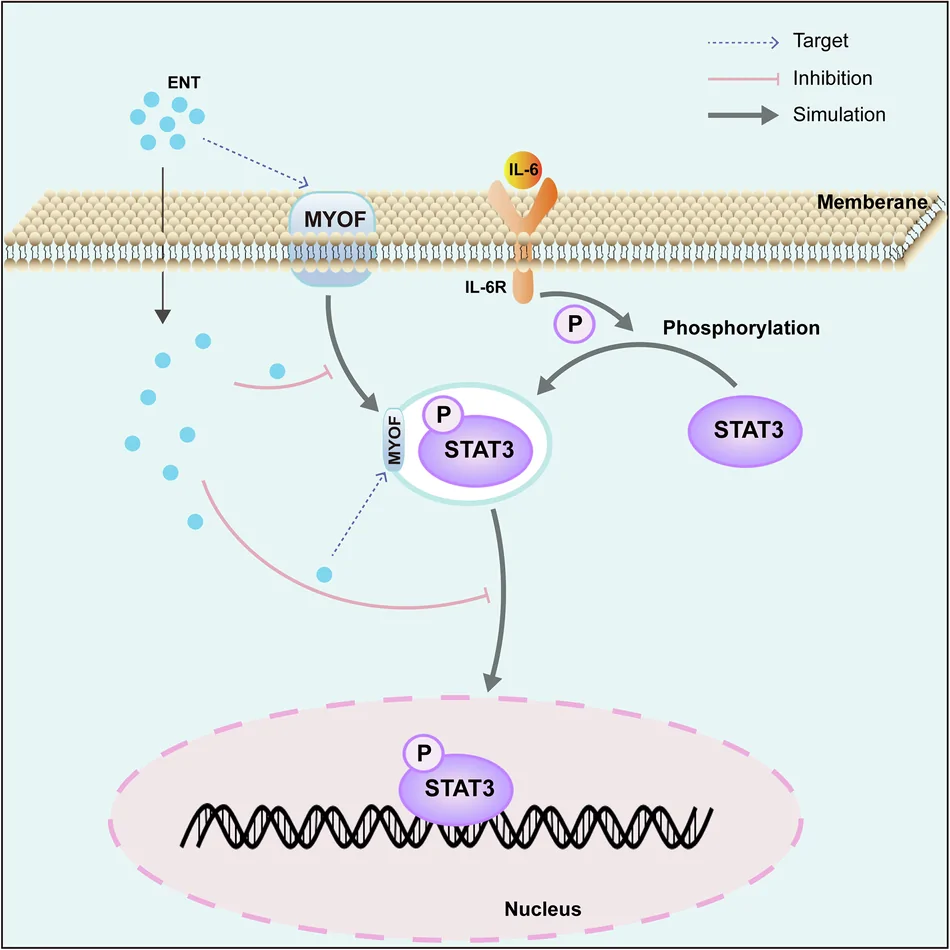
The diagram illustrates a novel regulatory mechanism of IL-6/STAT3 signaling involving MYOF. Upon IL-6 stimulation, STAT3 undergoes phosphorylation. MYOF then binds to phosphorylated STAT3 and facilitates its translocation into the nucleus to regulate target gene expression. The compound ENT acts as a targeted inhibitor of this process by binding to MYOF, thereby blocking the nuclear transport of phosphorylated STAT3 and suppressing downstream signaling.

The diagram illustrates a novel regulatory mechanism of IL-6/STAT3 signaling involving MYOF. Upon IL-6 stimulation, STAT3 undergoes phosphorylation. MYOF then binds to phosphorylated STAT3 and facilitates its translocation into the nucleus to regulate target gene expression. The compound ENT acts as a targeted inhibitor of this process by binding to MYOF, thereby blocking the nuclear transport of phosphorylated STAT3 and suppressing downstream signaling.

## Introduction

Glioblastoma (GBM) remains one of the most significant challenges in neuro-oncology. Despite extensive efforts, GBM continues to be a highly aggressive malignancy for which current standard-of-care treatments offer only limited improvements in long-term survival. Following the 2021 WHO classification update, diffuse gliomas are now categorized with a strong emphasis on molecular features, including IDH mutations and 1p/19q co-deletions. Notably, although most adult GBMs are IDH wild-type, nearly 70% of cases still have an “unknown” genetic status, indicating substantial gaps in our understanding of the disease [1–3]. The incidence of GBM continues to rise despite advances in surgical resection, combination therapy with temozolomide (TMZ), and the development of technologies that improve treatment precision. Due to the highly invasive nature of GBM, complete surgical resection remains unattainable, and the median overall survival of patients with GBM remains less than two years [4–6]. Recent advances in gene-targeted delivery systems and intraoperative imaging technologies have enabled more precise targeting of GBM cells; nevertheless, many of these innovations have not translated into significant improvements in patient outcomes [7–10]. Consequently, there is growing interest in identifying novel molecular targets and developing more effective therapeutic strategies for glioma treatment.

Myoferlin (MYOF), a member of the ferlin protein family, plays an important role in regulating membrane trafficking dynamics, membrane fusion, and membrane repair [11, 12]. Previous studies have shown that MYOF is overexpressed in multiple malignancies, including breast, lung, and colorectal cancers, and its elevated expression is generally associated with poor patient prognosis. In colon cancer, MYOF knockdown has been reported to enhance oxidative phosphorylation while suppressing tumor cell proliferation. However, despite these findings, the specific role of MYOF in GBM remains poorly understood [13–16].

Herein, we comprehensively investigated the role of MYOF in several GBM cell lines, including U251, U87, and LN229. Our findings demonstrated that MYOF is not merely passively expressed in GBM cells, but actively promotes tumor proliferation and invasion. Mechanistically, MYOF appears to facilitate the nuclear translocation of phosphorylated STAT3 (p-STAT3), a key signaling event associated with tumor progression. To identify potential therapeutic agents capable of disrupting this pathway, we performed a high-throughput screening of bioactive compounds and identified entacapone (ENT) as a promising candidate. Further analyses revealed that ENT effectively disrupts the interaction between MYOF and p-STAT3. Both in vitro and in vivo experiments confirmed that ENT suppresses glioma growth, suggesting its potential therapeutic value in GBM treatment. Collectively, our findings identify MYOF as a novel oncogenic factor in glioblastoma and highlight ENT as a potential therapeutic agent for targeting the MYOF/p-STAT3 signaling axis in this highly aggressive disease.

## Results

### MYOF is identified as a novel therapeutic target in GBM

**Fig. 1:**
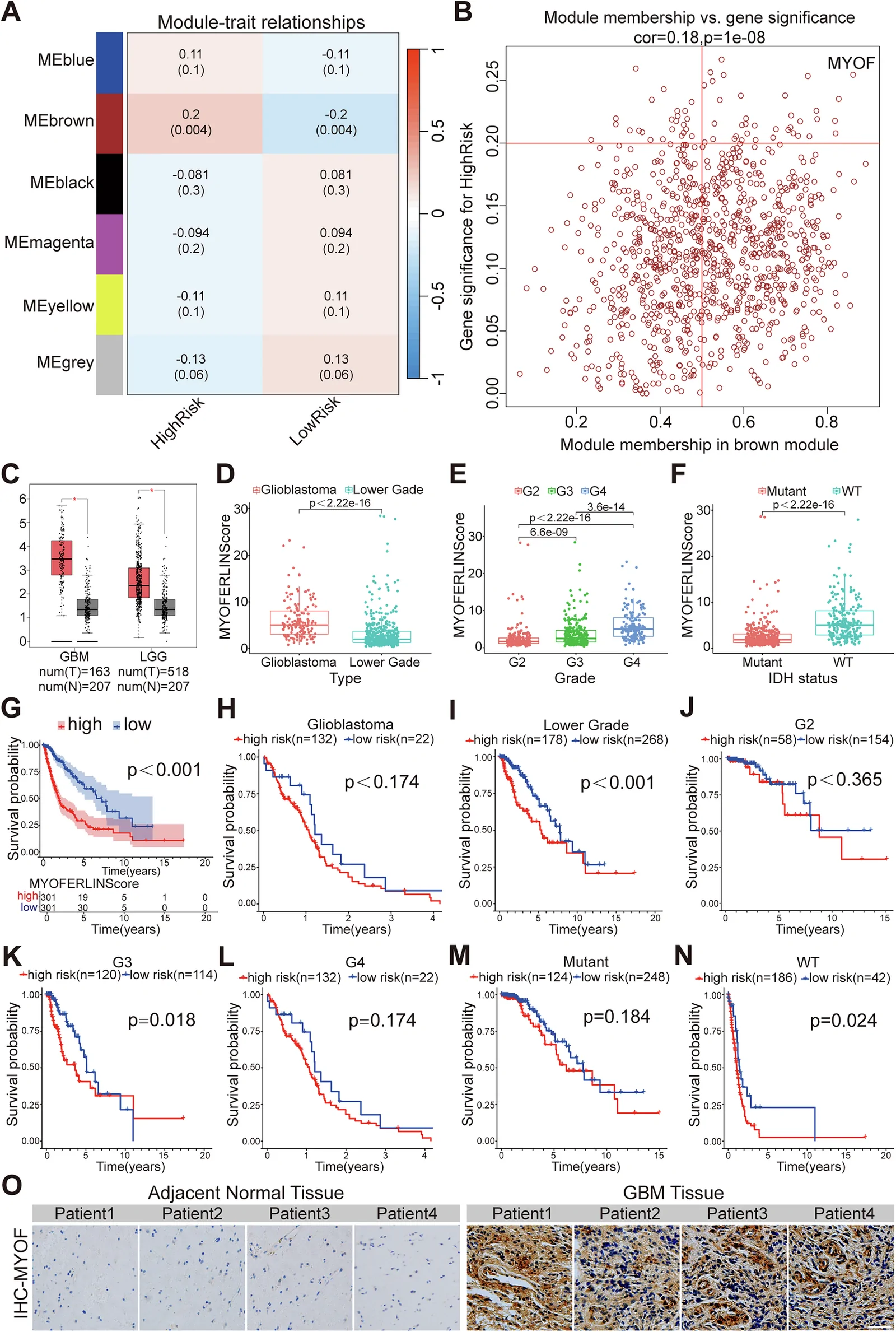
MYOF is identified as a novel therapeutic target in GBM. **A** The heatmap shows the correlation between the characteristic genes of each module and the phenotypic traits. The redder the color, the stronger the positive correlation. A *p*-value < 0.05 is considered statistically significant. **B** The MM and GS of the key brown module are positively correlated with the genes. MYOF, which is the most obvious gene, was selected. **C** The expression of MYOF in patients with GBM or LGG compared with normal from GEPIA. **D**, **E** MYOFERLIN score in patients with different types (Glioblastoma and Lower grade) and grade (G2, G3 and G4). **F** The relationship between MYOFERLIN score and IDH status. **G** The survival probability of patients with high or low MYOFERLIN score. **H**–**L** The survival probability of patients with different types (Glioblastoma and Lower grade) and grade (G2, G3 and G4). **M**, **N** The survival probability related with IDH status of patients with high or low MYOFERLIN score. **O** Immunohistochemical staining of MYOF, (200×).

To systematically identify co-expressed gene modules associated with specific biological processes, we performed weighted gene co-expression network analysis (WGCNA) using RNA-seq expression data. An unsupervised clustering approach was applied to construct a network comprising multiple gene co-expression modules. After evaluating different soft-thresholding powers, we selected β = 6 as the optimal parameter, as the scale-free topology fitting index (R²) reached 0.8 at this threshold, indicating a satisfactory fit to a scale-free network topology. Using the constructed network, we subsequently applied a dynamic tree-cutting algorithm to identify six major gene modules, each containing from dozens to thousands of genes. We further performed correlation analyses between module eigengenes and patient survival time. Notably, the brown module demonstrated a significant positive correlation with tumor stage (r = 0.2, *P* < 0.005), suggesting that this module may play an important role in biological processes associated with tumor progression and clinical outcomes (Fig. 1A). Among the six significant modules, 34 core hub genes within the brown module were identified based on the criteria of gene significance (GS > 0.2) and module membership (MM > 0.5). Notably, MYOF exhibited the highest module membership (MM = 0.878) and gene significance (GS = 0.25) values (Fig. 1B), supporting its potential central role within this module.

To validate these initial observations, we analyzed data from GEPIA, which confirmed that MYOF mRNA expression levels were significantly elevated in both GBM and LGG tissues compared with normal brain tissues (*P* < 0.01) (Fig. 1C). Consistent with these findings, analysis of the TCGA cohort demonstrated that MYOF expression was not only upregulated in gliomas overall, but was also significantly higher in GBM than in LGG (Fig. 1D). Furthermore, MYOF expression showed a positive correlation with tumor grade (Fig. 1E) and IDH mutation status (Fig. 1F). Survival analyses further revealed that elevated MYOF expression was associated with poorer overall survival (*P* < 0.01), with this association being particularly pronounced in patients with LGG (*P* < 0.01) (Fig. 1G–I). Although no statistically significant difference in survival was observed in the GBM-only cohort, the prognostic impact of MYOF expression remained evident in other subgroups. For example, patients with Grade 3 gliomas and high MYOF expression exhibited significantly poorer survival outcomes (*P* < 0.05; Fig. 1J–L). A similar trend toward worse prognosis was also observed in patients with wild-type gliomas (*P* < 0.05; Fig. 1M, N). To further validate these findings beyond bioinformatic analyses, we examined MYOF expression at the protein level. Immunohistochemical (IHC) staining was performed on tumor and adjacent normal tissues obtained from ten patients. Consistent with our genomic analyses, MYOF protein expression was markedly elevated in glioma tissues compared with adjacent normal tissues, providing direct experimental confirmation of MYOF overexpression in glioma samples (Figs. 1O, S1A).

### MYOF knockdown suppresses glioblastoma tumorigenicity

**Fig. 2:**
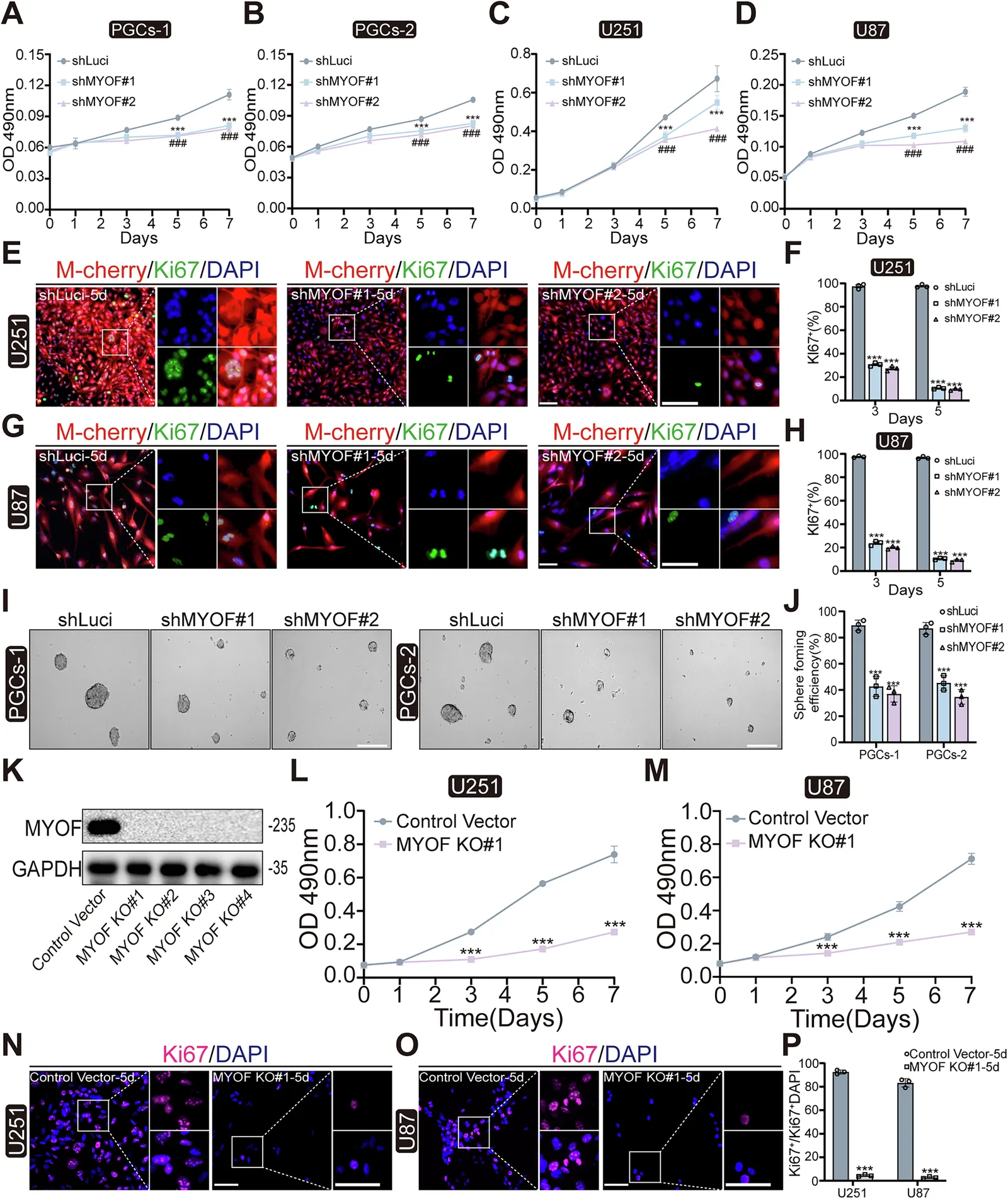
MYOF knockdown suppresses glioblastoma tumorigenicity. Data from two different shRNA knockout lines (called shMYOF#1 and shMYOF#2) were shown in comparison with scrambled shRNA called shLuci. Two primary glioma cell lines (**A**, **B**), U251(**C**) and U87 (**D**) were used to the assay. The absorbance was measured at 490 nm using a microplate reader on day 1, 3, 5 and 7. “*” represented the comparison between shMYOF#1 and shLuci while “#” represented the comparison between shMYOF#2 and shLuci (***/###p < 0.001). **E**–**H** The proliferation detected by immunofluorescence staining experiment of U251 and U87 human glioma cells transfected with shLuci and shMYOF lentivirus in the fifth day. All images were taken with fluorescence microscope. More than 90% of U251 (**F**), U87 (**H**) were unable to express KI67 (KI67^+^ (%) = KI67^+^ M-cherry^+/^M-cherry^+;^ M-cherry^+^ cells =154^-^567 for each condition). **I** The proliferation of primary glioma cells (PGCs) measured by Spheroidization experiment. **J** Statistical analysis of the number of cell spheroids in (**I**) (***p < 0.001). **K** Knockout efficiency of four different sgMYOF lentiviruses detected by Western blot in U251 cells. MTT assay of Control Vector and MYOF KO#1, U251 (**L**) and U87 (**M**) were used in this assay. **N**–**P** The Ki67 IF staining of U251 and U87 human glioma cells transfected with Control Vector and MYOF KO#1 lentivirus on the fifth day. The data are presented as mean ± SD. *P < 0.05, **P < 0.01, ***P < 0.001; one-way ANOVA, three independent experiments. Scale: 100 µm.

In the current study, we provide compelling evidence that MYOF is not merely a bystander, but rather a key driver of GBM aggressiveness by promoting both tumor cell proliferation and invasion. To determine whether GBM cells depend on MYOF for growth, we first silenced MYOF expression using two independent shRNAs (Fig. S2A–C). MTT assays demonstrated that MYOF knockdown markedly reduced cell viability, and this inhibitory effect became progressively more pronounced over time (Fig. 2A–D, S2D). Ki67 immunofluorescence further strengthened these findings, demonstrating a significant reduction in proliferating cells following MYOF depletion (Figs. 2E–H, S2E–H). To assess the clinical relevance of this observation, we next evaluated primary glioma cells, where MYOF loss markedly impaired tumor sphere formation (Fig. 2I, J). To ensure the robustness of our functional experiments, we further generated a CRISPR/Cas9-mediated MYOF knockout clone. After screening four candidate clones by WB to identify an optimal knockout line (Fig. 2K), we confirmed that MYOF ablation consistently suppressed proliferation across multiple GBM cell lines (Figs. 2L–P, S2I–K). Collectively, these cross- validated results provide strong evidence that MYOF is a potential therapeutic target in GBM.

Beyond promoting oncogenic proliferation, our findings suggest that MYOF also regulates cell cycle progression and metastatic potential. Following MYOF depletion, flow cytometry analysis revealed a marked accumulation of cells in the G0/G1 phase across multiple GBM models, indicating a significant cell cycle arrest at this checkpoint (Fig. S3A–F). Importantly, this growth inhibition was accompanied by a shift toward programmed cell death. TUNEL staining confirmed a substantial increase in apoptotic activity after MYOF knockdown (Fig. S3G–L), indicating enhanced induction of apoptosis. Together, these results suggest a dual effect of MYOF depletion, first inducing cell cycle arrest, followed by increased apoptotic cell death. We next examined how MYOF influences GBM cell migration and invasion, key hallmarks of glioblastoma aggressiveness. In transwell invasion assays, MYOF knockdown reduced invasive capacity by up to 70% (Fig. S4A, B), while wound-healing assays confirmed a marked reduction in migratory ability (Fig. S4C–F). Notably, a clear dose-response relationship was observed, as complete CRISPR-mediated knockout of MYOF impaired invasion more strongly than partial knockdown (Fig. S4G, H). Collectively, these findings identify MYOF as a central promoter of GBM dissemination. Rather than serving as a passive biomarker, MYOF appears to function as an active driver of malignancy, underscoring its potential as a therapeutic target in glioblastoma.

### MYOF knockdown suppresses tumor growth in an intracranial glioma model

**Fig. 3:**
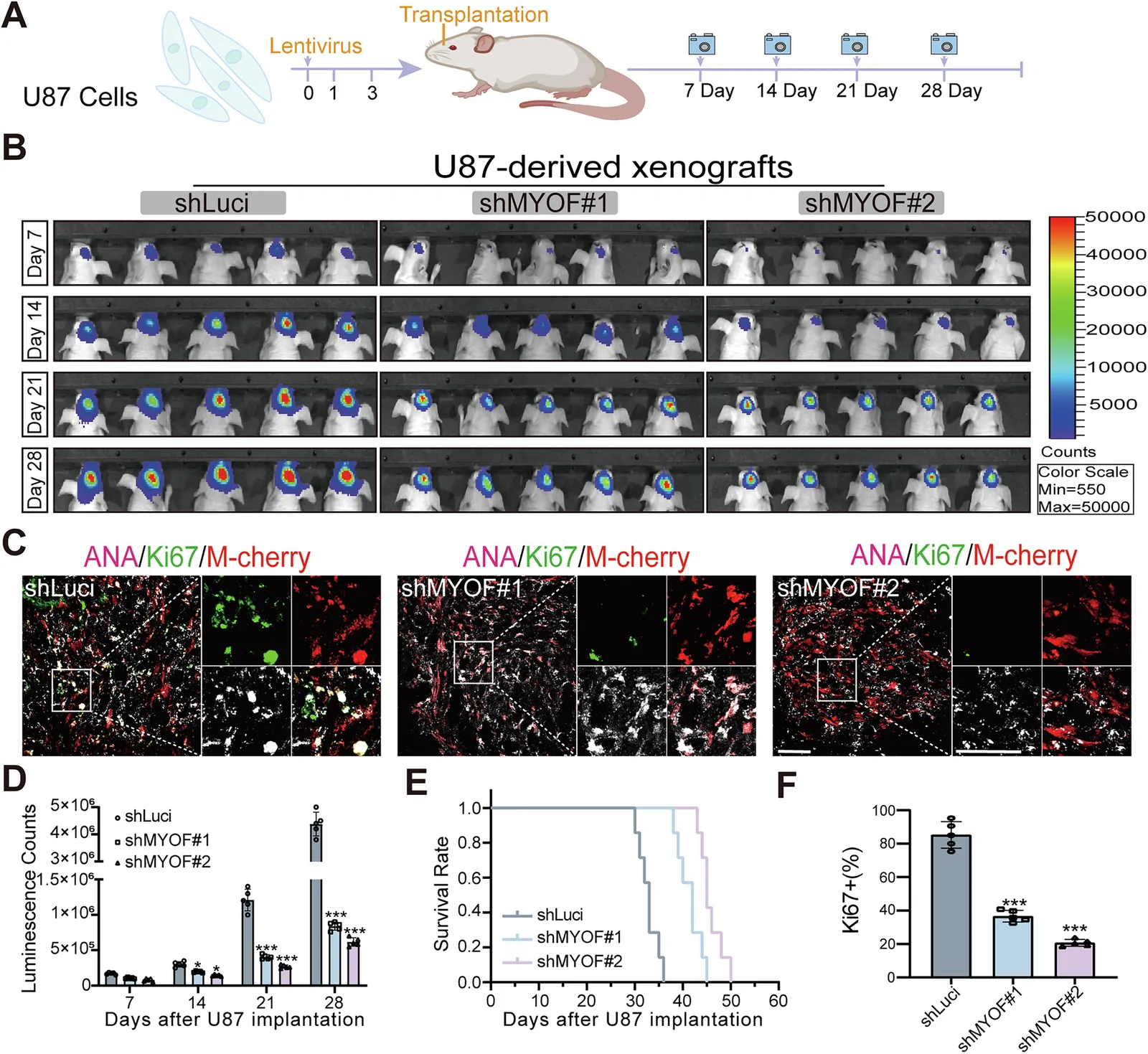
MYOF knockdown suppresses tumor growth in an intracranial glioma model. **A** The experimental scheme of orthotopic cell transplantation. **B**, **D** In vivo bioluminescence images and quantification of U87-derived xenografts (*n* = 5). **E** Kaplan-Meier survival analysis of transplanted mice after imaging (*n* = 5, *P* < 0.01 using log-rank test). **C**, **F** Lack of Ki67 marker of MYOF knockout U87 cells in xenografts (KI67+ (%) = KI67 + M-cherry+/M-cherry+). The data are presented as mean ± SD. ***P < 0.001 vs. shLuci group. ANA antinuclear antibody (Human). Scale: 100 µm.

To assess the in vivo effects of MYOF knockdown, U87 glioma cells were transduced with three lentiviral constructs: shLuci (control), shMYOF#1, and shMYOF#2. A luciferase reporter gene was also introduced to enable in vivo cell tracking and longitudinal bioluminescence imaging (Fig. 3A). Intracranial tumor implantation was performed in 35 nude mice, which were subsequently monitored weekly using a live-animal bioluminescence imaging system. As shown in Fig. 3B, D, tumors derived from MYOF-deficient cells exhibited significantly slower growth compared with control tumors. In addition to reduced tumor growth rates, mice in the shMYOF#1 and shMYOF#2 groups exhibited significantly prolonged survival compared with the control group (Fig. 3E). These in vivo findings were consistent with the in vitro results. Furthermore, immunohistochemical analysis demonstrated a pronounced anti-proliferative effect of MYOF knockdown, as reflected by reduced Ki-67 expression in shMYOF-treated tumors (Fig. 3C). The proportion of Ki-67–positive cells in tumors derived from MYOF-deficient mice was approximately 20–30% (Fig. 3F). Collectively, these results indicate that MYOF depletion suppresses glioma cell proliferation and slows tumor progression in vivo, supporting MYOF as a potential therapeutic target in glioblastoma.

### MYOF promotes glioma progression by facilitating nuclear transport of phosphorylated STAT3

Typically, STAT3 resides in the cytoplasm until phosphorylation at Tyr705 within its SH2 domain activates it, effectively switching it into a transcriptionally active state [17]. In malignant tumors, sustained hyperactivation of the STAT3 signaling pathway is a key driver of cell survival, angiogenesis, and invasion [18–20]. Once phosphorylated, STAT3 forms dimers that translocate into the nucleus, where it initiates the transcription of genes involved in processes ranging from differentiation to metastasis [21]. In gliomas specifically, the presence of p-STAT3 is often a critical determinant of tumor progression and patient prognosis [22–25]. Notably, MYOF is known to regulate cytoplasmic protein trafficking, potentially in cooperation with EH-domain-containing protein 2 (EHD2) [26]. Given that MYOF and p-STAT3 are frequently observed together in the nucleus during activation of IL6/STAT3 signaling, we surmised that MYOF may function as a carrier or facilitator of p-STAT3 nuclear translocation [11].

**Fig. 4:**
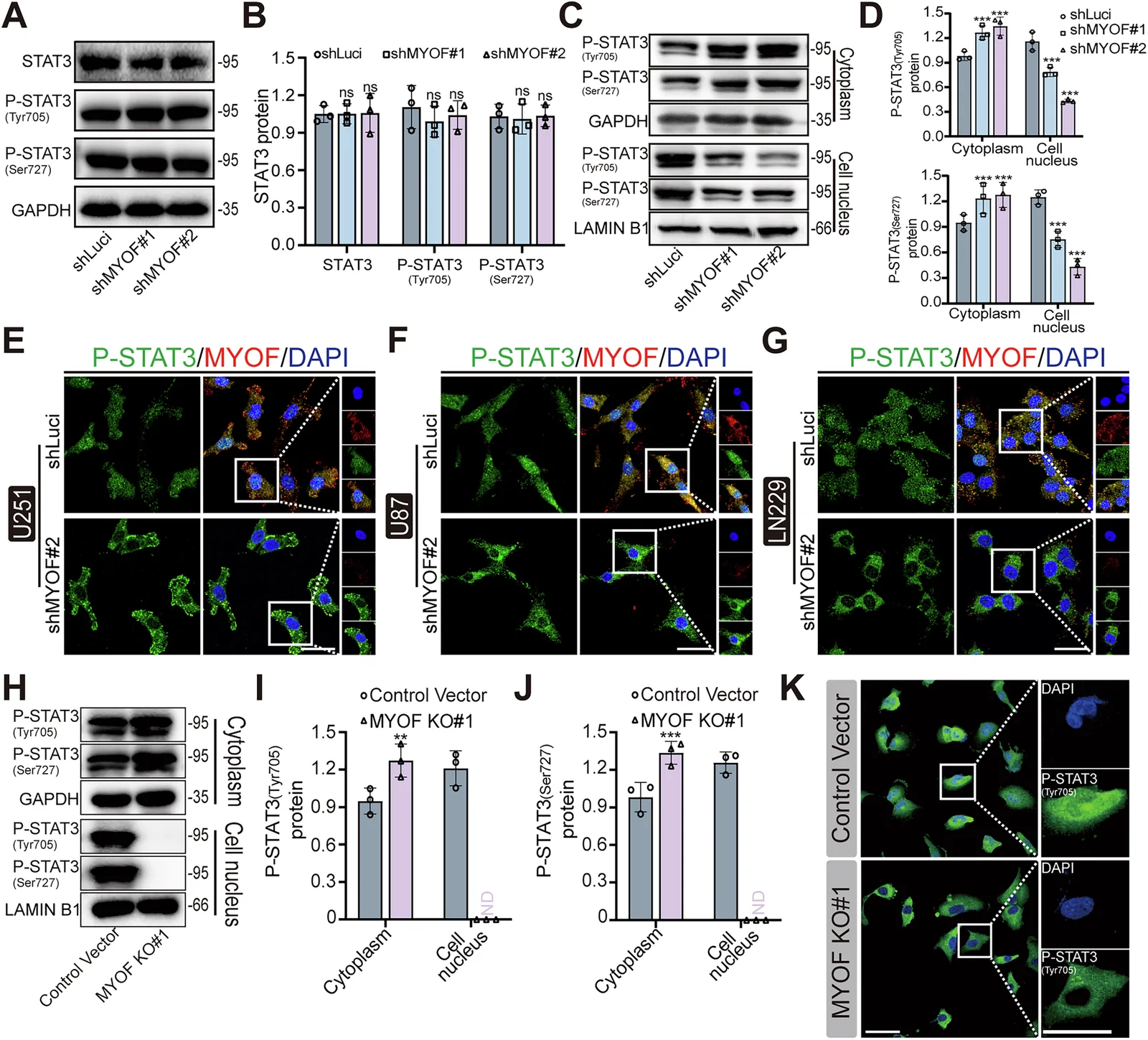
MYOF promotes glioma progression by facilitating nuclear transport of phosphorylated STAT3. **A**, **B** The total expression of STAT3, P-STAT3(Tyr705) and P-STAT3(Ser727) protein in U251 cells after MYOF knockdown. **C**, **D** The expression of P-STAT3(Tyr705) and P-STAT3(Ser727) in and out of the cell nucleus after MYOF knockdown detected by Western blotting assay (***P < 0.001). **E**–**G** The distribution of P-STAT3 in MYOF down-regulated cells detected by Immunofluorescence staining. The cells were stained on the fifth day with immunofluorescence and then photographed with a fluorescence microscope. **H**–**J** The expression of P-STAT3(Tyr705) and P-STAT3(Ser727) in and out of cell nucleus after MYOF knockout detected by Western blotting assay. **K** The distribution of P-STAT3 in MYOF knockout cells detected by Immunofluorescence staining. The cells were stained on the fifth day with immunofluorescence and then were photographed with a fluorescence microscope. The data are presented as mean ± SD. *P < 0.05, **P < 0.01, ***P < 0.001; ND not detected, one-way ANOVA, three independent experiments. Scale: 100 µm.

To test this hypothesis, we combined Western blot (WB) and immunofluorescence (IF) analyses. First, WB results showed that MYOF knockdown did not alter the total cellular levels of p-STAT3 (Tyr705 or Ser727) (Fig. 4A, B). However, a pronounced redistribution of p-STAT3 was observed: nuclear p-STAT3 levels were significantly reduced, while cytoplasmic levels were correspondingly increased (Fig. 4C, D). Consistently, IF imaging confirmed this redistribution, revealing that p-STAT3 was largely retained in the cytoplasm in MYOF-deficient cells (Fig. 4E–G). To validate these findings, we repeated the same experiments in CRISPR-engineered MYOF-knockout U251 cells, which similarly demonstrated that loss of MYOF effectively prevented nuclear accumulation of p-STAT3 (Fig. 4H–K). Collectively, these results suggest that MYOF is a critical mediator of p-STAT3 nuclear translocation, thereby contributing to the proliferation and progression of glioma cells.

### MYOF facilitates glioma progression by enhancing p-STAT3 nuclear transport following IL-6 stimulation

Recent studies have highlighted MYOF as a potential chaperone in the IL-6/STAT3 signaling pathway, an axis frequently hyperactivated in malignant tumors. In resting cells, MYOF is sequestered and maintained in an inactive state through interaction with EHD2. However, upon IL-6 binding to its receptor (IL-6R) and subsequent STAT3 phosphorylation, MYOF dissociates from EHD2, enabling it to participate in downstream signaling events within this pathway [11, 27, 28].

**Fig. 5:**
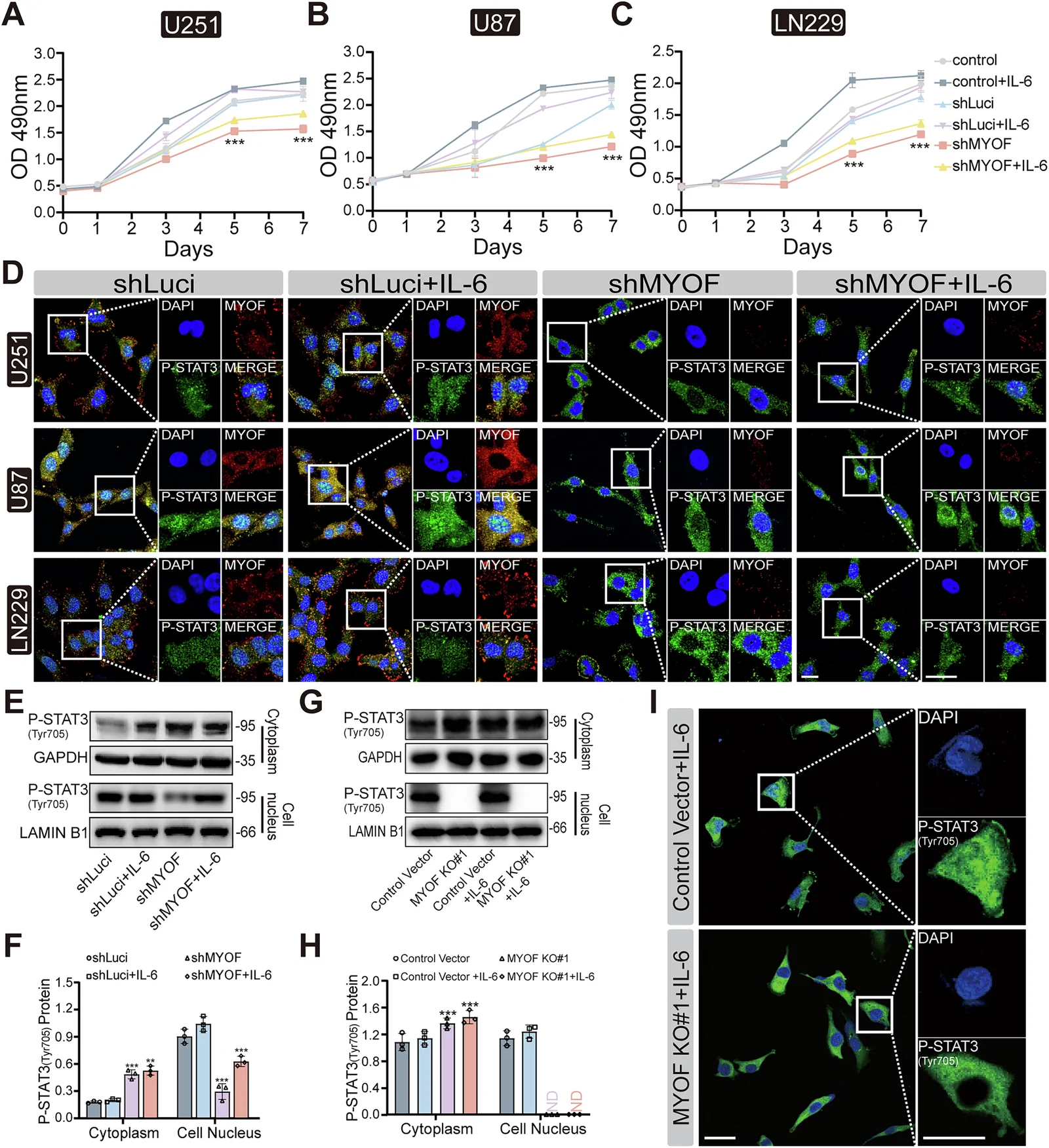
MYOF facilitates glioma progression by enhancing p-STAT3 nuclear transport following IL-6 stimulation. **A**–**C** The activity of MYOF down-regulated cells after IL-6 simulation tested by MTT assay. The glioma cells were firstly treated with lentivirus which carried MYOF down-regulated plasmid. Then, 100 μg /ml IL-6 was used to simulate p-STAT3 production in the first 24 h of MTT experiment (***P < 0.001). **D** The distribution of p-STAT3 in MYOF down-regulated cells treated with IL-6 observed by Immunofluorescence staining. **E**, **F** Western blotting assay was used to verify difference of p-STAT3(Tyr705) distribution in and out of MYOF down-regulated cells’ nucleus after IL-6 simulation (***P < 0.001). **G**, **H** The expression of P-STAT3(Tyr705) protein in and out of MYOF knockout cells’ nucleus after IL-6 simulation. **I** The distribution of P-STAT3(Tyr705) detected by Immunofluorescence staining in MYOF knockout cells after IL-6 treated. The data are presented as mean ± SD. *P < 0.05, **P < 0.01, ***P < 0.001; one-way ANOVA, three independent experiments. Scale: 100 µm.

To evaluate whether this MYOF-dependent mechanism contributes to glioma progression, we treated cells with an optimized dose of IL-6 (100 μg/mL for 24 h). In the control and shLuci groups, IL-6 produced the expected pro-survival effect, significantly enhancing cell viability. Although MYOF-depleted cells still responded to IL-6, the increase in viability was markedly attenuated compared with controls (Fig. 5A–C). Notably, IF and Western blot analyses of STAT3 localization revealed a clear mechanistic difference. In control cells, IL-6 induced a robust nuclear accumulation of p-STAT3 (Fig. 5D–F), consistent with the increased cell viability observed in the MTT assays. However, in MYOF-knockout U251 cells, this signaling axis was effectively disrupted. Despite IL-6 stimulation, p-STAT3 remained predominantly cytoplasmic and failed to translocate into the nucleus (Fig. 5G–I). Together, these results indicate a functional impairment in p-STAT3 nuclear transport in the absence of MYOF. Although IL-6 normally promotes STAT3 activation and nuclear translocation to drive glioma cell growth, this process is largely abrogated upon MYOF depletion. Collectively, these findings support the conclusion that MYOF is a key facilitator of p-STAT3 nuclear import, positioning it as an important regulator of IL-6/STAT3 signaling and, consequently, glioma malignancy.

### Targeting MYOF with small-molecule compounds effectively suppresses GBM cell growth

Small molecules and their applications in drug development and disease treatment continue to advance across multiple disciplines. In particular, the integration of high-throughput screening (HTS) with structural biology now enables rapid identification and targeting of specific small molecules for biological applications [29]. In recent years, numerous small-molecule agents have been developed for cancer treatment, including compounds designed to enhance anti-tumor immune responses and improve the body’s ability to combat malignancy [30–35].

**Fig. 6:**
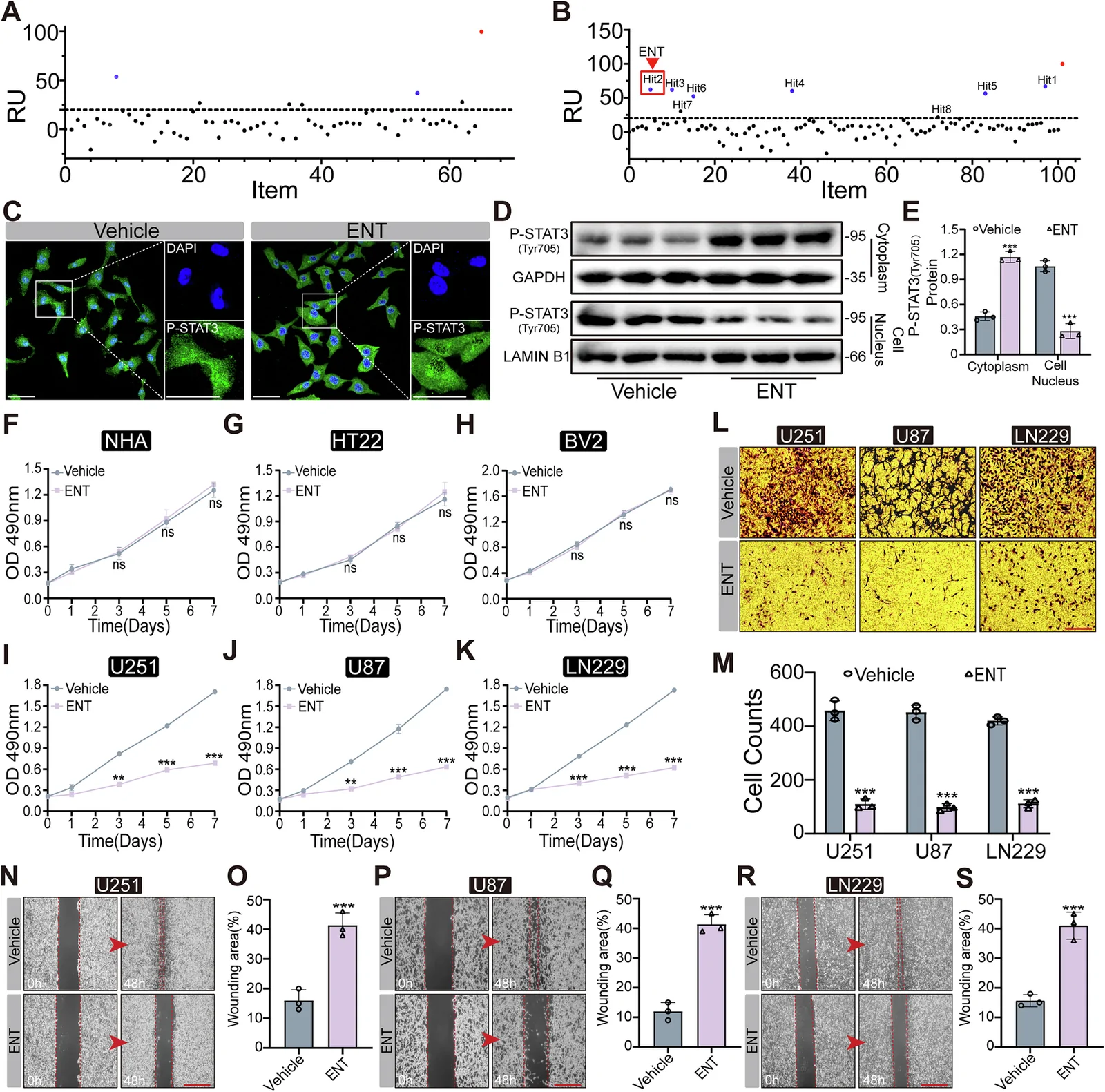
Targeting MYOF with small molecule compounds effectively suppresses GBM cell growth. **A** The first round involved group APR single-concentration screening. **B** The second round of individual SPR single-concentration screening. **C** Distribution of P-STAT3 in U251 cells after treatment with ENT through immunofluorescence experiments. **D**, **E** The expression of P-STAT3(Tyr705) protein in the cytoplasm and nucleus after treatment with ENT. **F**–**K** Changes in the proliferation ability detected by MTT assay of GBM cells (U251, U87 and LN229) and non-tumor cells (NHA, HT22 and BV2) after treatment with ENT. **L**, **M** Verify the effect of ENT treatment on the invasion ability of GBM cells through transwell experiments. **N**–**S** Examine the impact of ENT treatment on the migration capacity of GBM cells using scratch assays. The data are presented as mean ± SD. *P < 0.05, **P < 0.01, ***P < 0.001; one-way ANOVA, three independent experiments. Scale: 100 µm.

Here, surface plasmon resonance (SPR) technology was employed to screen for MYOF-targeting compounds by assessing their binding affinity to MYOF. The initial group-based single-concentration screening (APR) is shown in Fig. 6A, where red dots represent the positive control, and the two groups with the highest response units (RUs) are indicated by blue dots (groups 1 and 2). These two groups were subsequently selected for individual SPR analysis in a second screening round. In this second round, eight small-molecule compounds were identified with RU values exceeding 20, as shown in Fig. 6B. Functional characterization and feasibility assessments were performed for all eight candidate compounds. Based on these results, Hit 2 (entacapone, ENT) was selected for further investigation due to its favorable pharmacological properties. To further evaluate the dynamic behavior and binding stability of the MYOF–ENT complex, MD simulations and binding free energy analyses were conducted. Several parameters were analyzed. First, RMSD and centroid evolution analysis (CEA) were used to assess structural changes in the complex over the simulation period. The RMSD of the ligand-binding site exhibited notable fluctuations during the initial 0–70 ns of the trajectory, which, in conjunction with CEA results, suggested conformational rearrangements within the binding pocket. After approximately 70 ns, the RMSD reached a plateau, indicating that the complex had equilibrated and that the protein-ligand interaction remained stable at the new binding conformation (Fig. S5A, B). Next, RMSF analysis was performed to identify flexible and rigid regions of the protein during the simulation. The results showed that residues in the vicinity of ENT (residues 80–100) exhibited relatively low flexibility, suggesting a stable interaction between ENT and the binding site (Fig. S5C). Hydrogen bond analysis further revealed that ENT formed a hydrogen bond with the side chain of Lys102 in MYOF (Fig. S5D), with the number of hydrogen bonds fluctuating between 0 and 3 throughout the simulation trajectory (Fig. S5E). Hydrophobic interactions were also observed, with Ile104, Val86, and Tyr101 contributing Pi-Sigma and Pi-Alkyl interactions with ENT, respectively (Fig. S5D). In addition, residues Asp90, Ala87, and Ser105 contributed van der Waals (VDW) interactions to ligand binding (Fig. S5D). MMPBSA-based binding free energy decomposition identified Leu107, Val86, Ala87, and Ala84 as key residues involved in small-molecule recognition by MYOF (Fig. S5F). Finally, VDW and electrostatic (ELE) interaction energies between ENT and MYOF were calculated to monitor changes in binding strength throughout the simulation, excluding solvation effects. The binding energy (VDW + ELE) gradually stabilized over the course of the simulation, further supporting the dynamic stability of the MYOF–ENT complex (Fig. S5G). To further validate direct binding between ENT and MYOF, a cellular thermal shift assay (CETSA) was performed. As shown in Fig. S6A, B, MYOF protein levels in the vehicle group began to decline sharply at 45 °C. In contrast, in the ENT-treated group, MYOF exhibited markedly increased thermal stability, with a noticeable reduction in protein levels only occurring at temperatures approaching 60 °C. Together, these results provide evidence that ENT directly binds to and stabilizes the MYOF protein.

To delineate how ENT interferes with p-STAT3 nuclear transport, we performed IF and WB analyses. The results (Fig. 6C–E) showed that ENT treatment significantly reduced p-STAT3 nuclear translocation in U251 cells compared with controls. Notably, although ENT did not completely abolish nuclear import, its inhibitory effect appeared stronger than that observed with MYOF knockdown alone. We further evaluated the selectivity of ENT using MTT assays across three GBM cell lines and three non-malignant cell lines. The results showed that while non-cancerous cells were largely unaffected, ENT significantly inhibited the proliferation of all three GBM cell lines (Fig. 6F–K). Moreover, the drug not only inhibited cell proliferation but also actively induced apoptosis in glioma cells (Fig. S6C, D). This anti-tumor effect extended to metastatic behaviors as well, as transwell and wound-healing assays demonstrated that ENT significantly impaired the invasive and migratory capacity of GBM cells (Fig. 6L–S).

To further validate our findings and strengthen causal inference, we generated a MYOF overexpression plasmid (Fig. S6E, F) and performed rescue experiments. The underlying rationale was that if ENT exerts its effects primarily through MYOF, then restoring MYOF expression should partially counteract ENT-induced phenotypes. MTT assays (Fig. S6G–I) showed that MYOF overexpression partially restored cell viability that had been suppressed by ENT treatment. A similar trend was observed in invasion assays (Fig. S6J–O), where re-expression of MYOF significantly rescued the invasive capacity of GBM cells. Consistent with these functional changes, WB (Fig. S6P, Q) and IF (Fig. S6R) analyses further demonstrated that MYOF overexpression in ENT-treated cells restored p-STAT3 nuclear translocation. Taken together, this rescue effect provides strong mechanistic evidence that ENT exerts its inhibitory effects primarily by disrupting the MYOF–p-STAT3 axis, supporting MYOF-targeting small molecules as a promising therapeutic strategy for glioblastoma.

### ENT suppresses tumor growth in an intracranial glioma model

**Fig. 7:**
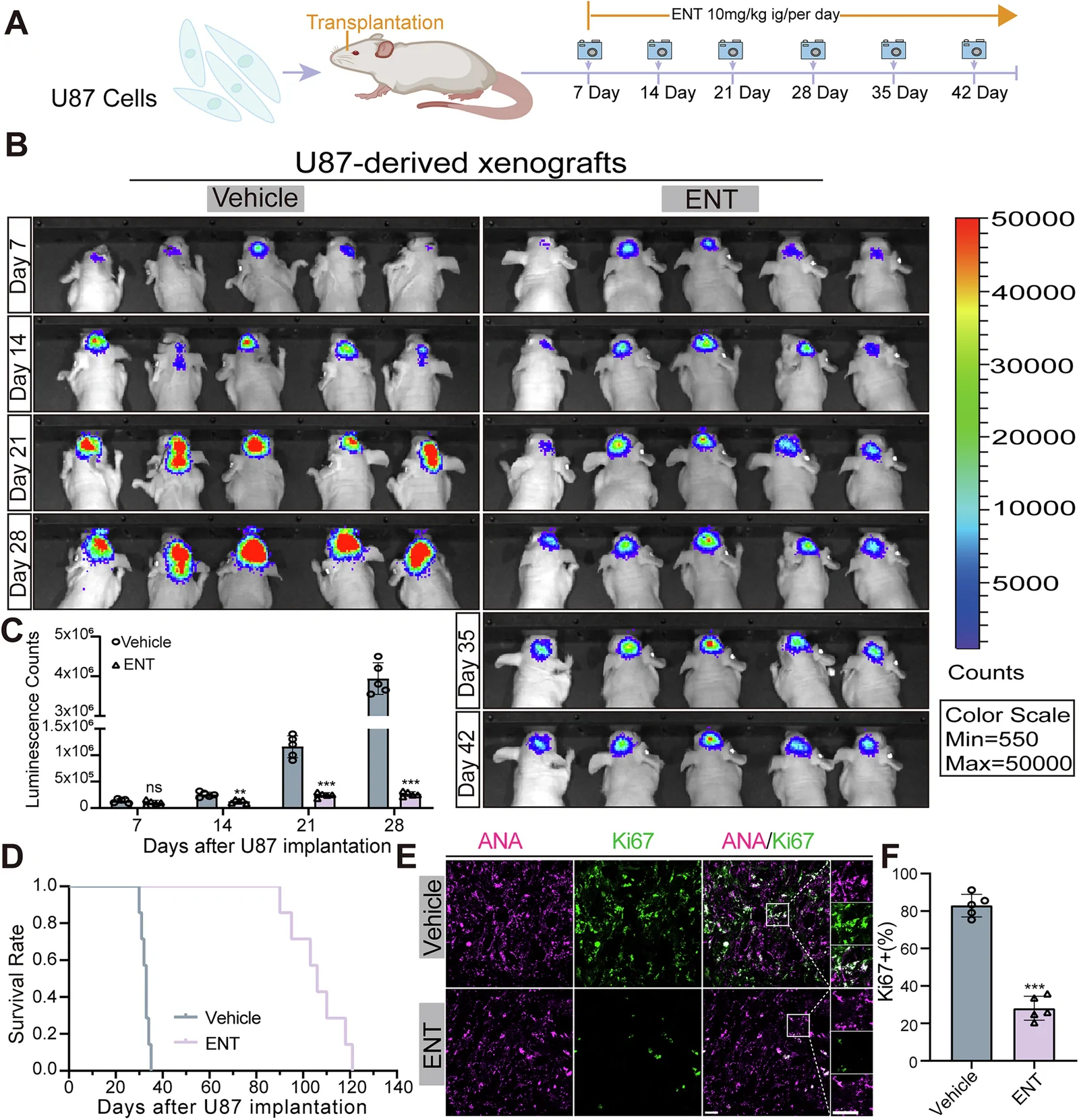
ENT suppresses tumor growth in an intracranial glioma model. **A** The experimental design of orthotopic cell transplantation. **B**, **C** In vivo bioluminescence images and quantification of U87-derived xenografts (*n* = 5). **D** Kaplan-Meier survival analysis of transplanted mice after imaging (*n* = 5, P < 0.01 using log-rank test). **E**, **F** Lack of Ki67 marker of ENT treated U87 cells in xenografts (KI67+ (%) = KI67 + M-cherry+/M-cherry+). The data are presented as mean ± SD. ***P < 0.001 vs. control vector group. ANA antinuclear antibody (Human). Scale: 100 µm.

Next, an intracranial xenograft model was established in nude mice to evaluate the in vivo efficacy of the small molecule ENT in inhibiting GBM growth. Following tumor implantation, mice were randomized into two groups and received once-daily oral administration of either phosphate-buffered saline (PBS) or ENT (10 mg/kg), starting on day 7 post-implantation (Fig. 7A). Bioluminescence imaging demonstrated that ENT treatment significantly reduced tumor burden compared with the PBS control group (Fig. 7B, C) and was associated with prolonged overall survival (Fig. 7D). All mice in the control group died within 30 days after tumor implantation, whereas mice in the ENT-treated group showed no mortality during the first three months post-implantation. In addition, Ki-67 immunohistochemical staining revealed significantly reduced Ki-67 expression in the ENT-treated group compared with controls (Fig. 7E, F), indicating suppressed tumor cell proliferation in vivo. Overall, these findings demonstrate that ENT not only inhibits GBM tumor growth in intracranial mouse models but also prolongs survival, supporting its potential as a therapeutic candidate for glioblastoma.

## Discussion

Despite advances in surgery, radiotherapy, and chemotherapy, GBM remains one of the most challenging malignancies in neuro-oncology, with recurrence being almost inevitable [36, 37]. In the present study, we applied WGCNA to RNA-seq data and identified six distinct gene modules. Notably, while the brown module was associated with tumor staging, MYOF emerged as a key hub gene within the brown module. Clinical data further demonstrated that high MYOF expression is strongly associated with advanced tumor stage, lymph node metastasis, and poor overall prognosis. Moreover, MYOF knockdown not only reduced GBM cell proliferation in vitro but also significantly suppressed xenograft tumor growth and prolonged survival in intracranial models. Mechanistically, MYOF depletion led to cytoplasmic accumulation of p-STAT3, a key mediator of oncogenic signaling. Lastly, we identified the small molecule ENT, which effectively inhibits glioma growth by targeting MYOF across multiple experimental systems.

MYOF, a member of the Ferlin family of transmembrane vesicle fusion proteins, has emerged as a key regulator of membrane dynamics, including transport, fusion, and repair [11, 12]. MYOF is upregulated in multiple tumor types, including breast, colon, melanoma, and lung cancers, and is associated with poor patient outcomes in GBM. In colon cancer cells, MYOF knockdown has been shown to enhance cellular respiration, resulting in reduced proliferation and increased cell death [13–16]. Despite these findings in colon cancer, the potential role of MYOF in the aggressive behavior of GBM has not been previously explored. Our results demonstrate that MYOF is significantly upregulated at both the mRNA and protein levels in GBM compared with normal brain tissue. Functionally, MYOF substantially contributes to GBM cell proliferation and invasion, as its knockdown reduces both processes in vitro and decreases tumor volume while prolonging survival in GBM xenograft models, indicating that MYOF may function as an oncogene in glioblastoma.

Interestingly, MYOF does not appear to alter the overall levels of STAT3 phosphorylation. Instead, it functions more like a chaperone or molecular escort, specifically facilitating the nuclear translocation of p-STAT3 [38–46]. Upon MYOF knockout, p-STAT3 is predominantly retained in the cytoplasm, thereby blocking downstream transcriptional programs that drive tumor growth. Of note, even after IL-6 stimulation, MYOF-deficient cells fail to fully restore STAT3 nuclear activity, further confirming that MYOF acts as a critical gatekeeper in this signaling pathway.

The future of oncology is shifting away from the “brute force” strategy of direct kinase inhibition, which has often shown limited success in clinical trials targeting STAT3. Instead, emerging approaches focus on more sophisticated ways to disrupt oncogenic signaling pathways, such as targeting upstream regulators or employing “molecular glues” to promote degradation of oncoproteins [47–49]. This evolving therapeutic paradigm is particularly important for challenging targets like STAT3, for which direct inhibition has historically demonstrated limited clinical efficacy. In addition, our data support the notion that the future of oncology will rely on developing small-molecule therapeutics that move beyond traditional direct kinase inhibition toward more refined strategies targeting upstream regulators in oncogenic pathways, such as MYOF. Using high-throughput screening, we identified the small molecule ENT, which modulates MYOF activity and inhibits p-STAT3 nuclear translocation. ENT further suppresses GBM cell proliferation, invasion, and migration while sparing most non-neoplastic cells. Together, these findings highlight the potential of ENT as a selective therapeutic candidate with promising translational value in glioblastoma.

Collectively, we are the first to identify MYOF as a pivotal regulator of STAT3 nuclear transport and a key driver of GBM progression. This study not only positions MYOF as a potential diagnostic biomarker but also put forwards ENT as a promising translational therapeutic agent. By bridging mechanistic insights with therapeutic application, our findings provide a potential framework for addressing the longstanding challenges in glioblastoma treatment.

## Conclusion

This research provides new information on how MYOF promotes the proliferation of glioblastomas through facilitating the transfer of phosphorylated STAT3 molecules from the cytoplasm into the nucleus. In addition, we have discovered that Entacapone (ENT), a drug originally approved to treat Parkinson’s Disease, inhibits MYOF. Therefore, developing ENT as an anti-cancer drug is a promising therapeutic option for treating glioblastoma. Nonetheless, further investigations are warranted to elucidate the underlying molecular mechanisms.

## Materials and methods

### Data download and standardization

The raw bulk-RNA-sequencing data of glioma were downloaded from the UCSC Xena (https://xena.ucsc.edu/) and NCBI GEO (https://www.ncbi.nlm.nih.gov/geo/) databases. There is a total of 162, 60 and 50 patients for the three datasets, respectively. The data were analyzed using SVA (3.38.0) and LIMMA (3.46.0) packages in R (4.0.3) to perform a batch normalization on the combined TCGA-GBM, GSE83300 and GSE74187 datasets. The SVA and LIMMA packages are BioConductor packages. TCGA and GEO provide RNA sequencing matrix files as well as clinical information relevant to the data. We then converted our probe IDs from R to the International Standards for Gene Values/Symbols using an annotation package for R. The expression data was normalized, and after normalization, the expression data is Log2 transformed. To analyze the expression of MYOF and its effect on the percentage of survival among GBM and LGG patients, we used the clinical and relative mRNA expression data from GEPIA (http://gepia.cancer-pku.cn/) and TCGAs.

### WGCNA and core gene selection

Weighted Gene Co-Expression Network Analysis (WGCNA), a type of “weighted” correlation network analysis, is a systems biology technique that enables the detection of connection patterns between multiple genes within the same biological sample but at different time points. In this research, we have assigned two different phenotypes to the combined survival duration of TCGA-GBM (the American College of Surgeons Cancer Committee’s GBM database), GSE83300, and GSE74187 datasets according to the median survival duration for each dataset. We used the WGCNA R package to examine the relationship between genes associated with the membrane proteins found in these datasets in order to identify sets of co-varying gene sets. We identified potential biomarker genes by analyzing the consistency among gene sets and the correlation of these gene sets to the defined phenotypes as defined above. The appropriate soft thresholding method (power) for the combined cohort was assessed using the “pickSoftThreshold” function and the resultant soft threshold value used in generating a hierarchical clustering dendrogram from the 1-TOM similarity matrix in order to evaluate the correlation between modules. The modules exhibiting the highest degrees of correlation with respect to the clustering coefficients were subsequently selected as candidates for further analysis based on their associations with respect to clinical characteristics.

### Cell culture and authentication

Cell lines U87 (RRID: CVCL_GP63), U251 (RRID: CVCL_0021), and LN229 (RRID: CVCL_0393) of human glioblastoma were acquired from the Cell Bank of the Chinese Academy of Sciences in China. Each of the aforementioned human glioma cell lines was verified by means of short tandem repeat (STR) analysis then matched up against existing databases. Contamination with Mycoplasma was routinely checked for using PCR methods. Cell lines of the human glioblastoma types U251, U87, and LN229, along with those of the human embryonic kidney (HEK) cell line HEK-293T (RRID: CVCL_0063), were grown according to manufacturer’s instructions in Dulbecco’s modified Eagle’s medium (DMEM; Gibco; 11995040) containing a supplement of 10% Fetal Bovine Serum (FBS; Gibco; 10099141), and Penicillin-Streptomycin (100 Unit/mL/100 µg/mL; Gibco; 15140122) at 37 °C under an atmosphere of 5% CO₂ in a rainfall developer.

### Plasmid and lentiviral vector construction

For plasmid construction, 97mer oligonucleotides (Table S1) manufactured by SANGON biotech (China) were amplified using a specific polymerase chain reaction (PCR) protocol, digested with a restriction endonuclease, inserted into a lentivirus vector containing m-cherry marker (NEB, USA), and then transformed into *E. coli* for further amplification. Then lentivirus vectors and packaging plasmid (pMDL, VSV-G, pRSV (RRID: Addgene_12253) were transfected into hek-293t cells, and the culture medium was changed after 12–16 h. The filtered supernatant, obtained at 24 and 48 h, was employed as the inoculum for cell infection [50]. In unbiased shRNA screening, lentiviruses (each candidate gene includes two shRNAs) were constructed and enumerated.

### CRISPR/Cas9 plasmid construction and lentiviral packaging

The lentiCRISPRv2 plasmid (Addgene #98292) was digested and dephosphorylated with FastDigest Esp3I at 37 °C for 30 min. The digestion product was purified by gel extraction (QIAquick Gel Extraction Kit) and eluted in 50 µL EB buffer. Successful Esp3I digestion was confirmed by the presence of a ~ 2 kb fragment; only the larger target band was excised and purified. Oligonucleotide pairs (Table S2) were phosphorylated and annealed in a thermal cycler (37 °C for 30 min, 95 °C for 5 min, followed by a ramp-down to 25 °C at 5 °C/min). Annealed oligos were diluted 1:200 in sterile water and ligated into the vector at room temperature for 1 h or at 4 °C overnight. Lentiviral particles were produced in HEK-293T cells via transfection with the CRISPR construct and packaging plasmids (pCMV-VSV-G, Addgene #8454; pGag-pol, Addgene #14887). Filtered supernatant collected 24 h post-medium change was used for subsequent cell infections.

### Real-time PCR (RT-PCR) and quantitative real-time PCR (qPCR)

Total RNA was extracted from U251 cells and pre-treated with shMYOF or shLuci using Trizol (15596018, Thermo Fisher Scientific, USA). For cDNA synthesis, 2 μg RNA per sample (*n* = 3) was reverse transcribed using Revert Aid First Strand cDNA Synthesis Kit (K1622, Thermo Fisher Scientific, USA). The real-time PCR (RT-PCR) primers are given in Table S3, as previously reported. RT-PCR was performed using Iraqi general SYBR Green supermix (1725124, Bio-Rad, USA). The cycle threshold (CT) values of the target gene were normalized to the cycle threshold of the internal control gene (GAPDH), and the relative change of gene expression was calculated using the formula 2^−ΔΔCt^.

### Protein extraction and western blot

The total protein, a complex of different molecules, was obtained from RIPA lysis buffer (89900, Thermo Scientific USA) and quantitatively assessed using the BCA protein analysis kit (23227, Thermo Scientific USA). The extraction provided a day-by-day protein profile (*n* = 3) via SDS-PAGE analysis followed by transfer onto a PVDF membrane. Two hours after blocking with 5% milk, membranes were exposed manually and then incubated with primary antibodies for 16 h (RRID: MYOF(DF13168), Affinity, USA; Phospho-STAT3, Y705, RRID: AB76315, Abcam, USA; and GAPDH, RRID: M20006, Abmart, USA) after which membranes were incubated with secondary antibody (RRID: S0001, Affinity, USA) for 2 h. Immunoreactive bands were quantified by Image J software.

### MTT assay

An MTT assay method was used to assess the ability of MYOF to inhibit the proliferation of U251, U87, LN229 and glioma stem cell proliferation. MYOF-knockdown and shLuci knockdown (control) cells were plated into 96-well plates (2000 cells/well) and incubated under standard culture environment (37 °C, 5% CO2) conditions for 4 h. After the incubation period, 50 µl of MTT reagent (10 mg/ml) was added to each well; then all 96-well plates were further incubated for 4 h at 37 °C, 5% CO_2_. After this second incubation period, the media was carefully removed and 150 µl of DMSO was added to solubilize the formazan crystals that had formed. The 96-well plates were gently shaken until complete dissolution occurred, and the absorbance of each well was recorded at 490 nm with a plate reader.

### Immunofluorescence (IF)

The nude mouse’s left ventricle was infused with 20 ml normal saline, followed by 20 ml 4% paraformaldehyde (PFA). The dehydrated brain tissue was then fixed and stored in liquid nitrogen. Glioblastoma cells and brain sections were fixed with 4% paraformaldehyde for 20 min and were treated with a PBST solution (0.4% Triton in PBS) with 5% BSA protein solution (ST025, Beyotime, China) for 30 min, to block non-specific staining. After this step, the brain sections were kept at 4 degrees Celsius overnight in the presence of Ki67 rabbit polyclonal antibody (RRID: AB15580, Abcam, USA) followed by introduction of secondary antibodies (RRID: AB150077, AB150115, Abcam, USA), kept at 37 °C for 1 h. The cell’s nucleus was counterstained with DAPI (RRID: AB285390, Abcam, USA). The confocal laser scanning microscope (Olympus, JPN) was used to view the ICF and IHF staining, while Image-J (RRID:SCR_003070) program was used for image analysis. To quantify the total number of cells counted, we used nine random fields of view from three different samples from five nude mice to obtain 45 random fields of view from three random sections from each nude mouse.

### Scratch wound healing assay

Cell migration was evaluated via a scratch wound assay. U251 and LN229 cells (shLuci- or shMYOF-treated) were cultured in 60-mm dishes at a density of 2.5 × 10^6 cells/well and cultured for 24 h to form a confluent monolayer. A straight scratch was made in each monolayer with a 200 μL pipette tip. Cells were then incubated in a serum-free medium (37 °C in 5% CO_2_); wound closure was monitored by periodic imaging with an inverted microscope, and wound area was measured until complete closure occluded.

### Transwell assay

Transwell chambers were utilized to determine the cell invasion capability. The upper chambers of the Transwell chambers received an initial 50 µL of Matrigel matrix. Following this preparation, 100 µL of either serum-free media, containing 5 × 10⁴ cells, was placed into each of the prepared upper chambers and 500 µL of DMEM + 10% fetal bovine serum was added to the lower chambers. Following a 48 h’ incubation period at 37 °C, 5% CO₂ environment, the cells that invaded through the Matrigel were fixed, using cold methanol, stained with crystal violet and then counted under a microscope.

### Primary glioblastoma cell culture and authentication

Patient-derived glioblastoma cells were established from freshly resected glioblastoma tissue obtained at The First Affiliated Hospital of Wenzhou Medical University (Wenzhou, China). All procedures were conducted with written informed consent from patients and approval from the hospital’s Institutional Ethics Committee. Tumor tissues were enzymatically and mechanically dissociated and cultured under stem cell–permissive conditions. Cell identity was confirmed by STR profiling, and tumor origin was validated by histopathology. Mycoplasma contamination was ruled out using PCR-based assays, and only early-passage cells (P3–P6) were used for downstream applications to preserve tumor heterogeneity.

Following surgical resection, tumor specimens were transferred to a sterile petri dish using autoclaved forceps and finely minced with a sterile razor blade. The tissue fragments were then washed into a 15 mL conical tube with 5 mL of freshly prepared Dissociation Medium 1 (4.75 mL Dissociation solution, 200 μL papain stock, and DNase I). The tube was incubated in a 37 °C water bath for 30 min, with manual mixing using a 5 mL pipette every 5 min. After dissociation, the pellet was sequentially resuspended and incubated in 5 mL of Dissociation Medium 2 (containing collagenase I, hyaluronidase, and trypsin) and 2 mL of Red Blood Cell Lysis Buffer (10 min at RT). The suspension was then diluted with 8 mL of wash buffer, filtered through 100 μm and 70 μm cell strainers, and finally resuspended in Tumor Sphere Culture Medium (DMEM/F-12 + GlutaMAX supplemented with amphotericin B, gentamicin, HEPES, and B27 minus vitamin A) [51].

### Animals and ethics consideration

Male BALB/c nude mice between the ages of 6 and 8 weeks (18–22 g) were sourced from Shanghai Charles River Experimental Animal Co., Ltd., located in Shanghai, China, and were continuously housed in an environment with free access to both food and water. The protocol for each experiment was reviewed and approved by Wenzhou Medical University’s Institutional Animal Care and Use Committee according to the National Institute of Health Guidelines for the Care and Use of Laboratory Animals.

### Orthotopic glioblastoma xenograft model

Mice were anesthetized with 2% isoflurane and positioned in a stereotactic frame (RWD Life Sciences). A burr hole was drilled at stereotactic coordinates (1 mm anterior, 2 mm lateral to bregma). Three days after lentiviral transduction, 5 × 10⁵ U87 cells in 5 µL were injected into the striatum at a depth of 3 mm. The burr hole was sealed with bone wax, and the scalp was sutured. Tumor growth was monitored weekly via bioluminescence imaging (IVIS Lumina X5; PerkinElmer) initiated 10 min after intraperitoneal fluorescein injection (10-s exposure) [52]. For survival analysis, a subset of mice (*n* = 5 per group) was monitored until natural death. At 28 days post-implantation, another subset (*n* = 5 per group) was euthanized for immunohistochemical analysis of tumor burden. All procedures, including group allocation, surgery, imaging, and data analysis, were performed in a randomized and blinded manner.

### Surface plasmon resonance (SPR) binding assay

SPR experiments were performed on a BIAcore T200 instrument at 25 °C using a CM5 sensor chip (GE Healthcare). A flow cell was activated with a 7-min injection of 200 mM EDC and 50 mM NHS at a flow rate of 10 µL/min. The target protein (in 10 mM sodium acetate, pH 5.0) was immobilized via two consecutive injections under the same flow conditions. Remaining reactive sites were blocked with 1 M ethanolamine. A reference flow cell was treated identically but immobilized with PBS (pH 5.0). Following equilibration with running buffer, serial dilutions of analyte were injected (150 s per cycle, 10 µL/min). The chip surface was regenerated between runs with 10 mM glycine-HCl (pH 2.0). Binding sensorgrams were obtained by subtracting reference cell responses and were globally fitted to a 1:1 Langmuir binding model using BIAcore T200 Evaluation software (v.2.0). Final binding curves were generated in Origin 7 (v.7.0552).

### Molecular dynamics (MD)

Molecular dynamics (MD) simulations were carried out with GROMACS 2022.2. The Amber14SB force field was used for the protein, and the TIP3P water model was applied throughout. Ligand parameters were prepared as follows: antechamber-generated AM1-BCC charges, and GAFF2 atom types were assigned to all ligand atoms. ACPYPE was then used to convert the ligand topology into GROMACS format. Sodium and chloride ion parameters were taken from the Joung-Cheatham set, which is compatible with TIP3P. The protein-ligand complex was placed in a dodecahedral box with a minimum distance of ≥1.2 nm from the protein surface to the box edge. The system was solvated with TIP3P water and brought to 0.15 M ionic strength using Na⁺/Cl⁻ counterions. Before production runs, the system was energy-minimized using the Steepest Descent method until the maximum force was below 1000 kJ mol⁻¹ nm⁻¹. Next, the system was equilibrated at 298 K—first under NVT for 200 ps, then under NPT for another 200 ps. The production run was performed under NPT conditions for 100 ns, using a 2-fs time step with the Verlet scheme. Long-range electrostatics were handled by PME, with a cutoff of 1.2 nm for both Coulomb and van der Waals interactions. Bond lengths involving hydrogen atoms were constrained with LINCS. Temperature was maintained at 298 K by Nose-Hoover coupling, while pressure was held at 1 bar using the Parrinello-Rahman method. Trajectory snapshots were saved every 10 ps. After the simulation, GROMACS utilities, VMD, and PyMOL were used for structural analysis. Binding free energy was calculated with gmx_MMPBSA.

### Cellular Thermal Shift Assay (CETSA)

GBM cells (U251) were cultured to 80% confluence and treated with either ENT or DMSO for 4 h. Following treatment, cells were harvested, washed twice with ice-cold PBS, and resuspended in PBS supplemented with a protease inhibitor cocktail to prevent protein degradation during the heating process. The cell suspension was divided into equal aliquots (50 µL each) into PCR tubes. These samples were then subjected to a thermal gradient using a precision thermocycler. The temperatures were set at 35, 40, 45, 50, 55, 60, 65, 70, and 75 °C, with each temperature point maintained for 3 min. Immediately following the heat challenge, the tubes were incubated at room temperature for 3 min to allow for protein equilibration. To recover the soluble protein fraction, the cells underwent three rapid freeze-thaw cycles using liquid nitrogen and a 37 °C water bath. The resulting lysates were centrifuged at 20,000 × *g* for 30 min at 4 °C to pellet the denatured and aggregated proteins. The supernatant, containing the stabilized soluble proteins, was carefully collected. The presence of MYOF in each fraction was then quantified via Western blot. The resulting band intensities were normalized against the lowest-temperature point (35 °C) to generate thermal denaturation curves, allowing us to evaluate the shift in melting temperature (Tm) induced by ENT binding.

### Statistical analysis

Data representation includes Mean ± Standard Deviation (SD), Statistical Analysis performed using GraphPad Prism, Differences between 2 Groups compared utilizing Mann-Whitney Test, Comparison among 3+ Groups compared utilizing One-Way ANOVA. Survival Curves were created (Kaplan-Meier) and compared (Log-rank (Mantel-Cox)). *P* < 0.05 is statistical significance.

## Supplementary information

Supplementary information

Full unedited blot images

## Data Availability

All data generated or analyzed during this study are available within the article and Supplementary Files. Additional supporting data are available from the corresponding authors upon reasonable request.
