# Supplementary material for: Uncovering MYOF as a novel therapeutic target in glioblastoma: mechanistic insights and drug discovery
Source: Cell Death Discov. 2026 Jul 14;12:409. doi: 10.1038/s41420-026-03254-0 (PMC13645784; doi:10.1038/s41420-026-03254-0)
Supplement: Supplementary file 1 [file 41420_2026_3254_MOESM1_ESM.pdf]

Supplementary Information

Uncovering MYOF as a Novel Therapeutic Target in Glioblastoma: Mechanistic Insights and Drug Discovery

Peiqi Zhao, Zhen Chen, Jiajie Zhu, Zibin Zhang, Zhenqiu Xing, Fan Tang, Kankai Wang5, Ying Zhang, Hong Chen, Qichuan Zhuge and Jianjing Yang

This file contains Supplementary Figures S1–S6 and Supplementary Tables S1–S3.

Supplementary Figure 1.

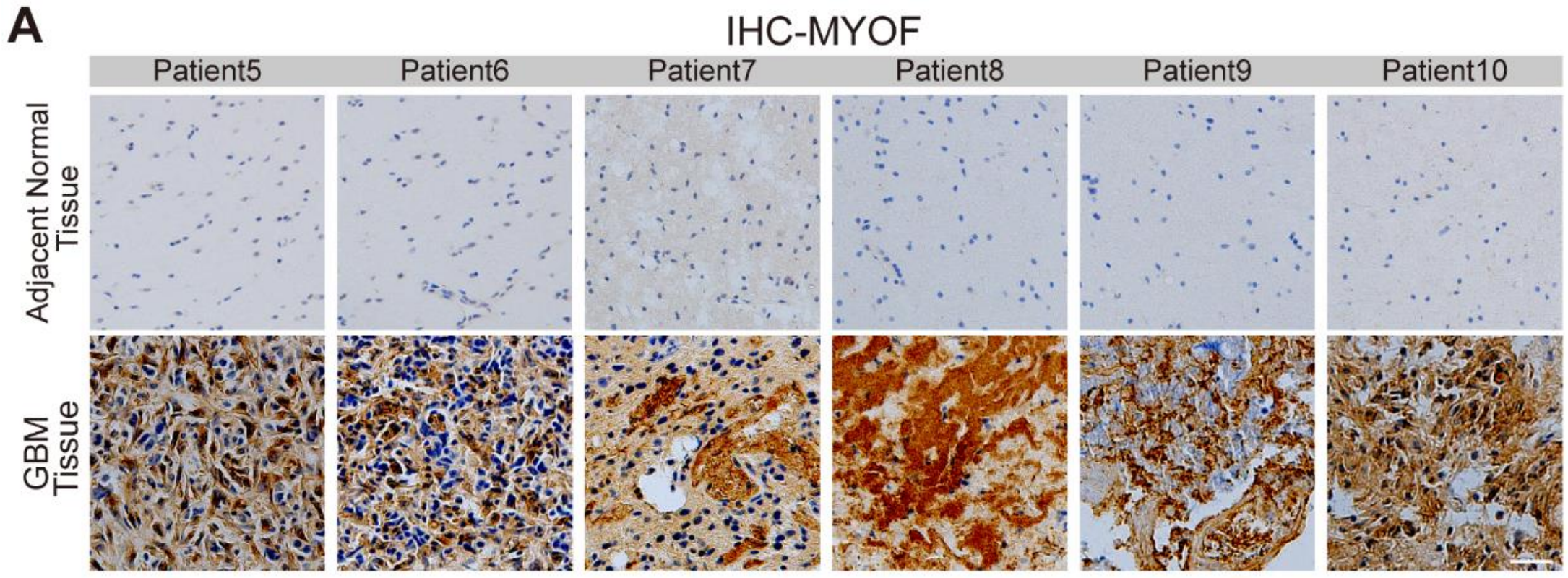

Figure S1. (A) Immunohistochemical staining of MYOF, (200x).

Supplementary Figure 2.

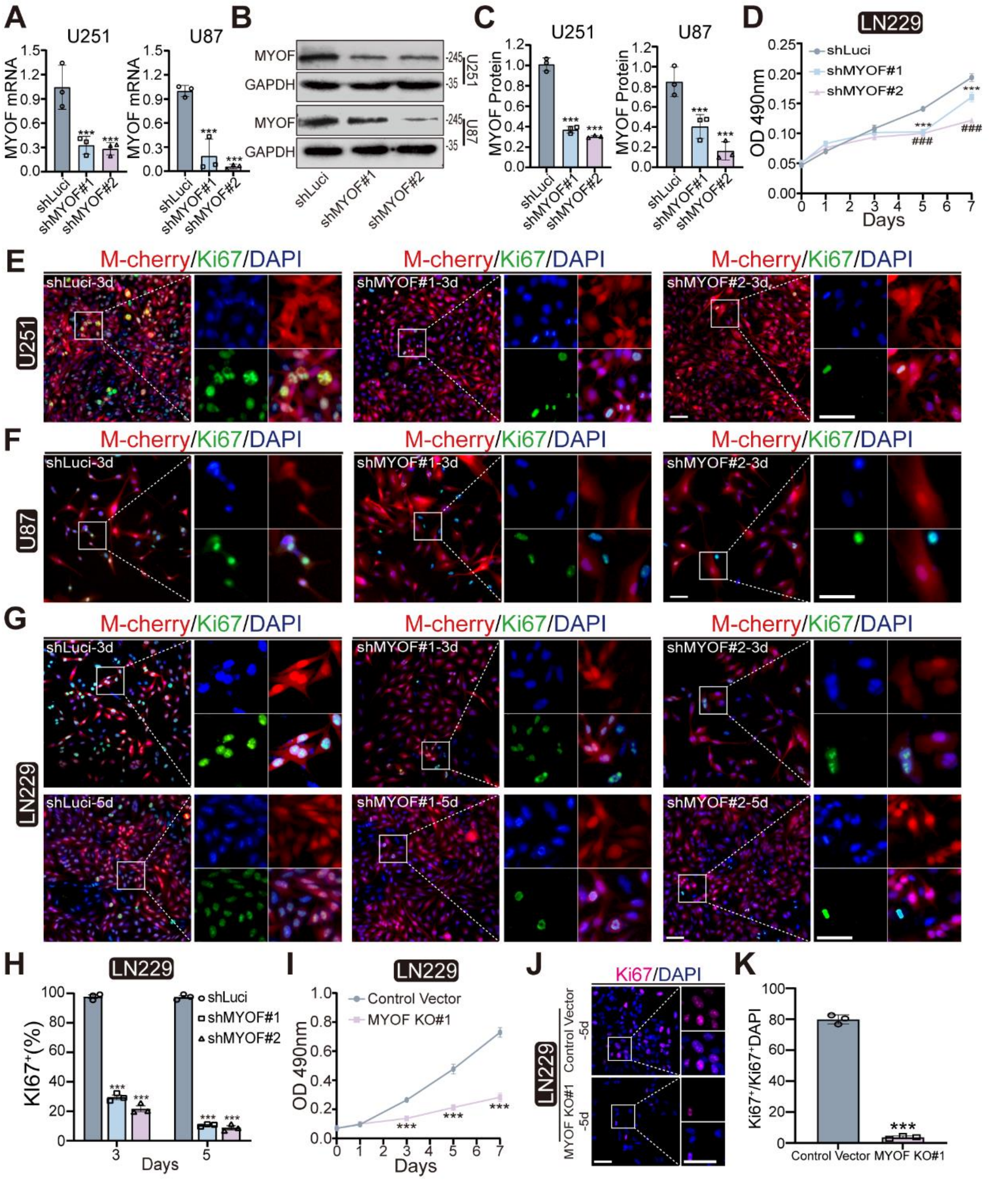

Figure S2. Knockdown of MYOF significantly attenuates the proliferative capacity of GBM cells. (A) Knockdown efficiency of MYOF mRNA levels in U251 and U87 cells. (B, C) Knockdown efficiency of MYOF protein in U251 and U87 cells. (D) MTT assay in LN299 cells with MYOF knockdown. (E, F) Immunofluorescence staining images of the KI67 indicator on the third day after U251 and U87 cells were infected with lentivirus. (G, H) Immunofluorescence staining of the KI67 indicator on the third and the fifth day after LN229

cells were infected with lentivirus. (I) MTT assay in LN299 cells with MYOF and knockout. “*” represented the comparison between shMYOF#1 and shLuci while “#” represented the comparison between shMYOF#2 and shLuci (***/###p<0.001). (J, K) Immunofluorescence staining images of the KI67 indicator on the fifth day after U251 were infected with lentivirus. The data are presented as mean ± SD. ***P < 0.001 vs. shLuci or control vector group. Scale: 100μm.

Supplementary Figure 3.

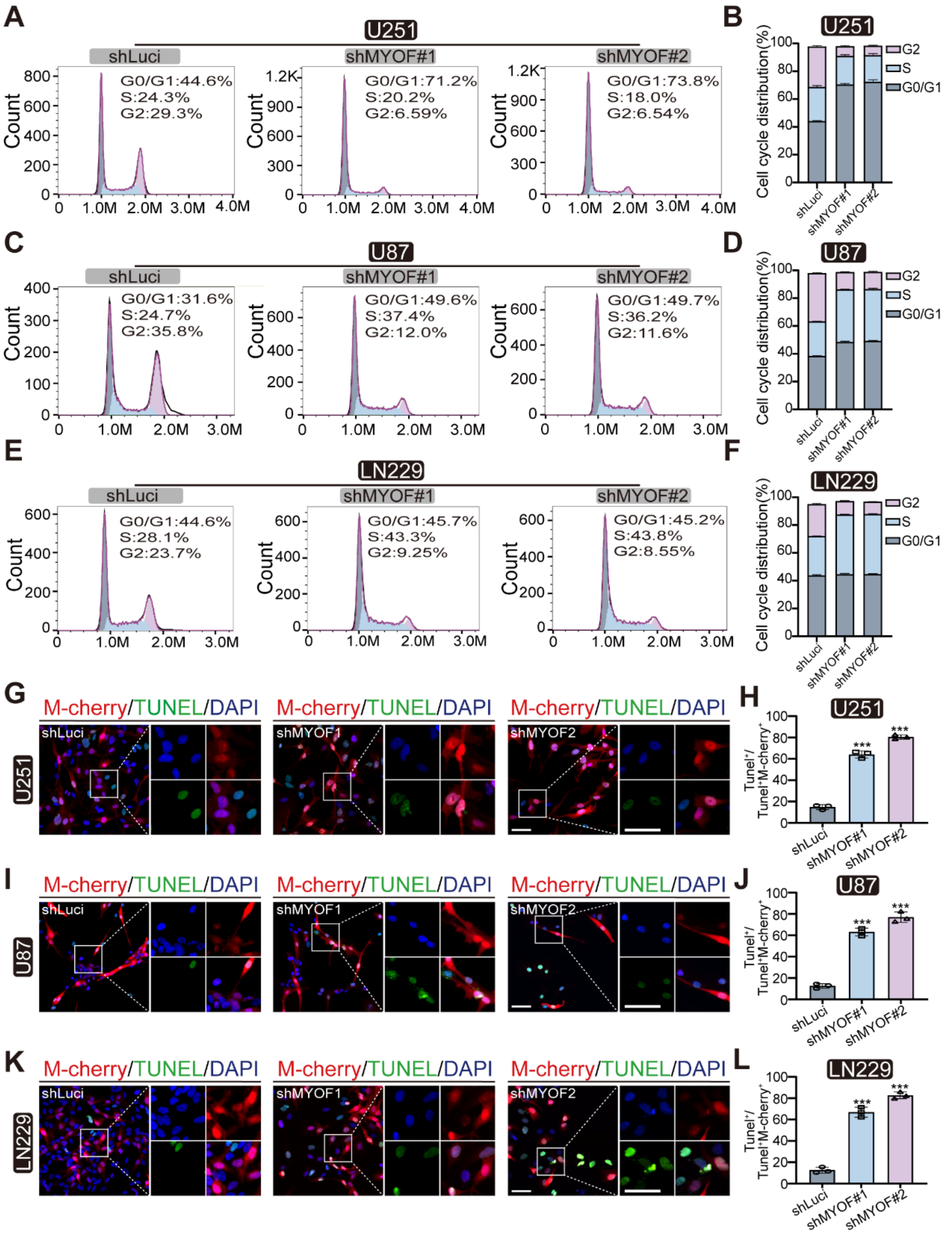

Figure S3. Knockdown of MYOF expression leads to reduced cell cycle progression and increased apoptosis in GBM cells. (A-F) Cell flow cytometry experiments to detect the cell cycle of GBM cells after knockdown of MYOF. Statistical analysis of cell cycle distribution in B, D, F (Pink refers to cells in G2 phase, blue refers to cells in S phase and

gray refers to cells in G1 phase) ($p<0.001$). (G-L) Validation of the effect of MYOF knockdown on U251 (G, H), U87 (I, J) and LN229 (K, L) cells via TUNEL staining. The data are presented as mean ± SD. ***$P < 0.001$ vs. shLuci group. Scale: 100μm.

Supplementary Figure 4.

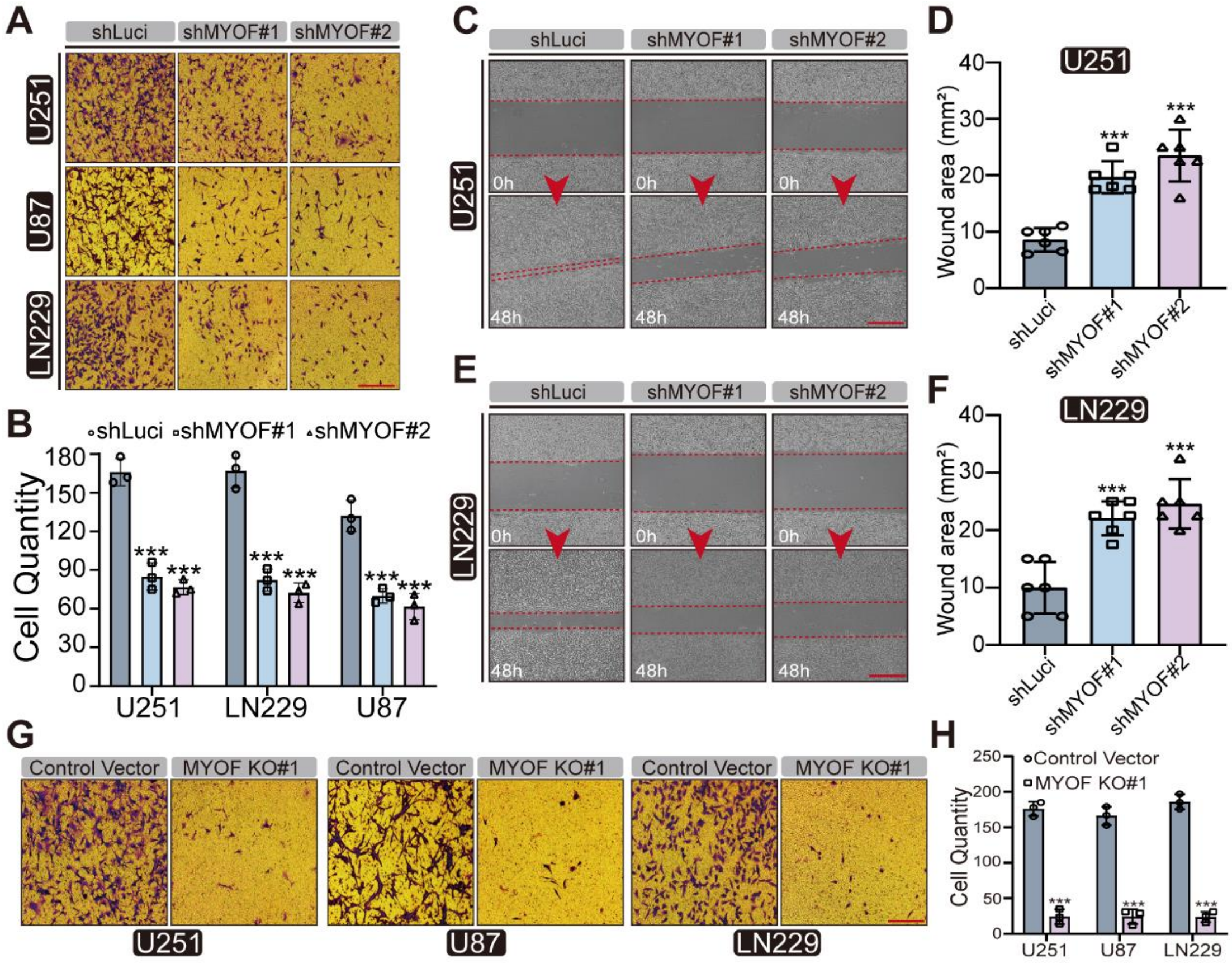

Figure S4. Knockdown or knockout of MYOF expression reduces the invasion and migration abilities of GBM cells. (A, B) Transwell experiments to validate the invasion ability of GBM cells after knockdown of MYOF. (C-F) Scratch assays to assess the migration ability of GBM cells after knockdown of MYOF. (G, H) Transwell experiments to validate the invasion ability of GBM cells after knockout of MYOF. The data are presented as mean ± SD. *P<0.05, **P<0.01, ***P<0.001; one-way ANOVA, three independent experiments. Scale: 100μm.

Supplementary Figure 5.

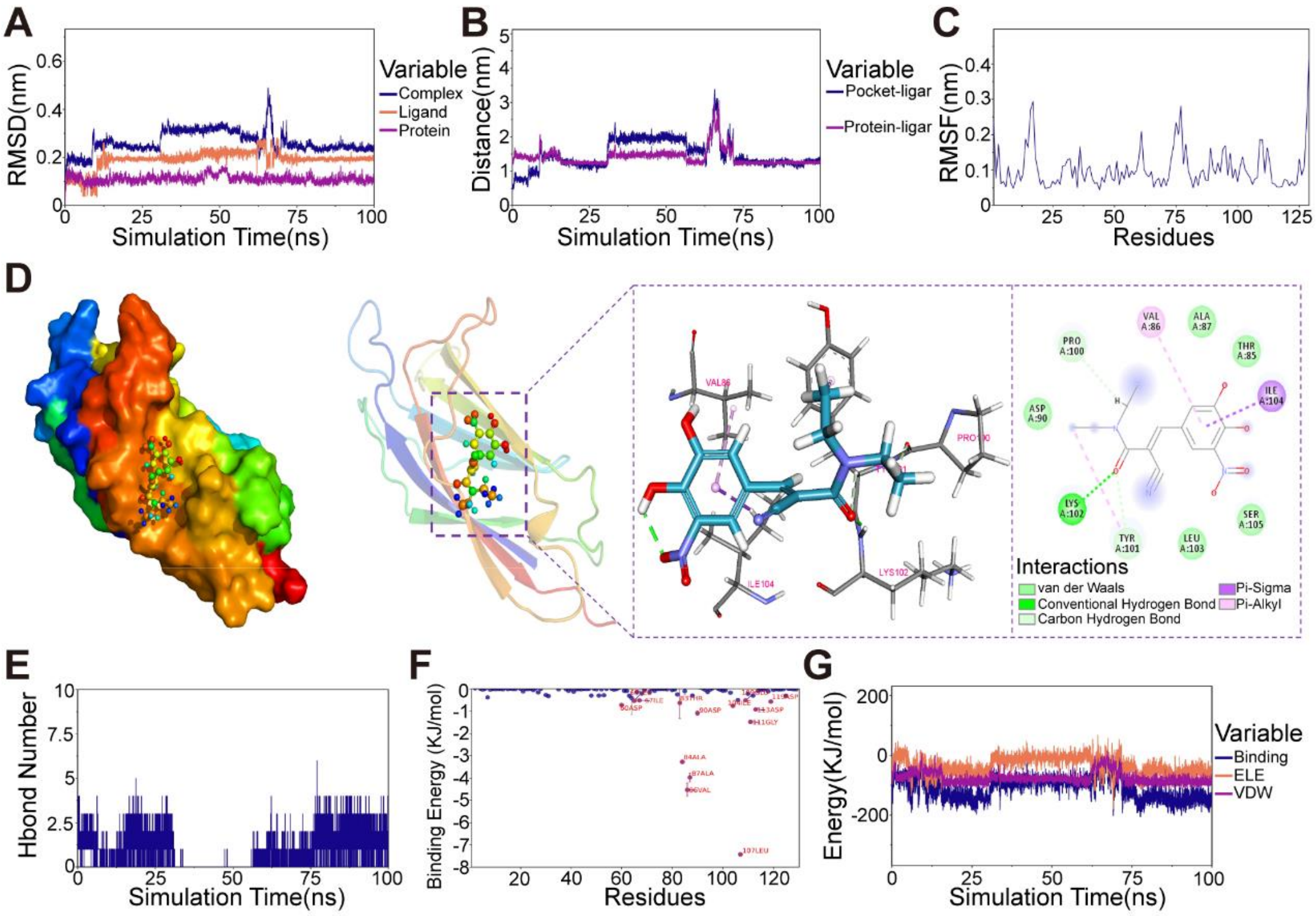

Figure S5. Molecular Dynamics (MD) Simulation and Binding Mode Analysis of the Protein-Ligand Complex. (A) Root Mean Square Deviation (RMSD): Time-dependent RMSD values for the complex (blue), ligand (orange), and protein backbone (purple) over a 100 ns simulation trajectory, indicating the structural stability and convergence of the system. (B) Distance Profile: Monitoring of the distance between the ligand and the binding pocket (blue) and the center of mass of the protein (purple) during the simulation. (C) Root Mean Square Fluctuation (RMSF): Local flexibility of individual protein residues. Peaks represent highly flexible loop regions, while troughs indicate stable secondary structures. (D) Binding Mode Visualization: * Left: Surface representation of the protein colored by hydrophobicity/domain with the ligand docked in the primary pocket. Center: Ribbon and stick representation highlighting the 3D spatial orientation of the ligand within the binding site. Right: 2D interaction diagram showing specific molecular contacts, including

conventional hydrogen bonds (green), carbon-hydrogen bonds (light green), Pi-Sigma (purple), and Pi-Alkyl interactions (pink). (E) Hydrogen Bond Analysis: The total number of hydrogen bonds formed between the ligand and the protein throughout the 100 ns MD trajectory. (F) Binding Free Energy Decomposition: Per-residue energy decomposition identifying key amino acids contributing to the binding affinity. Major contributors include LEU107, VAL86, and ALA87. (G) Energy Components: Evolution of the total binding energy (dark blue) over time, partitioned into electrostatic (ELE, orange) and van der Waals (VDW, purple) components.

Supplementary Figure 6.

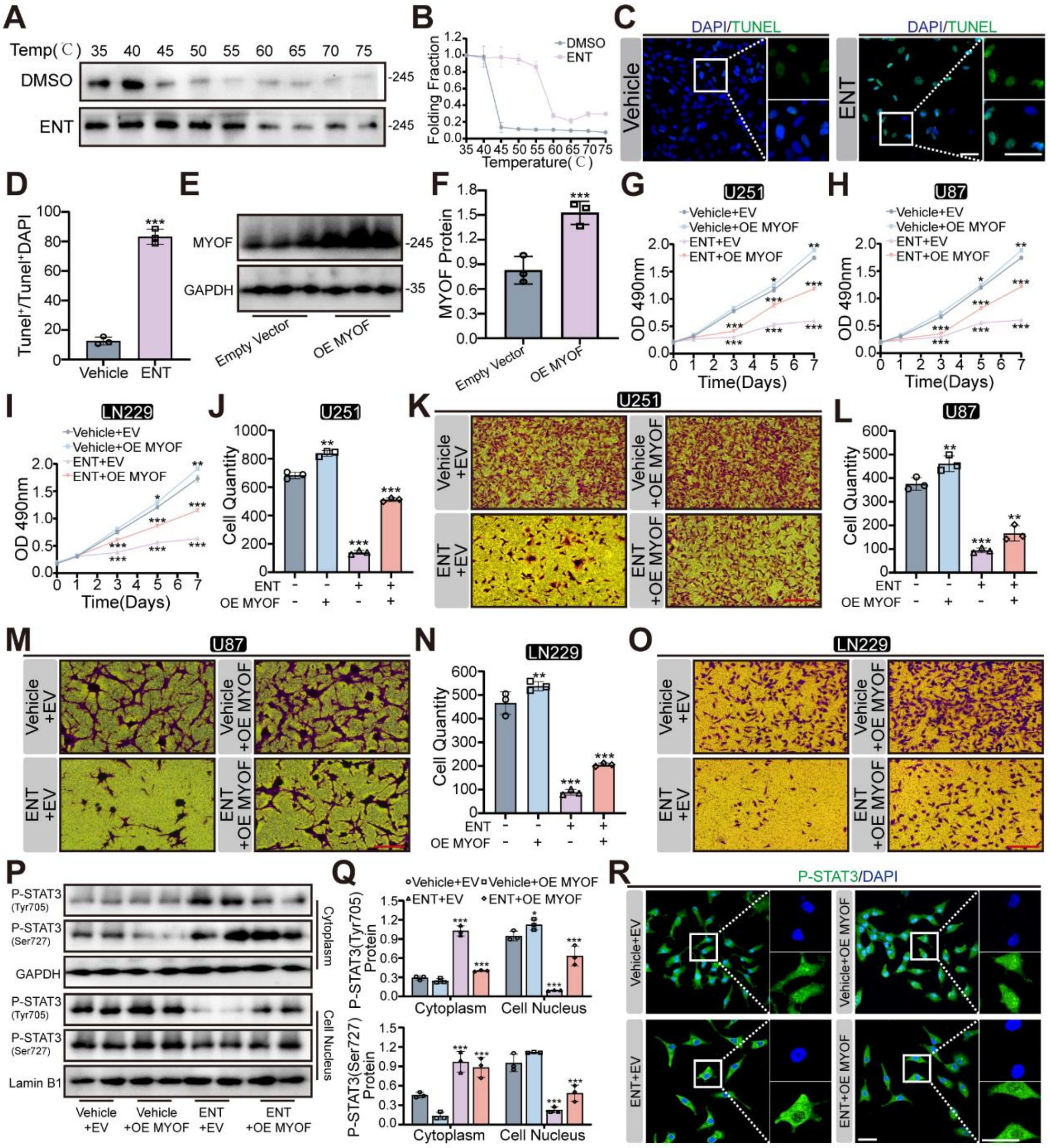

Figure S6. ENT targets MYOF to inhibit glioblastoma progression via the STAT3 signaling pathway. (A, B) Cellular Thermal Shift Assay (CETSA): Representative WB (A) and corresponding thermal aggregation curves (B) showing the thermal stability of MYOF in the presence of DMSO or ENT across a temperature range of 35–75°C. ENT significantly increases the melting temperature (Tm) of MYOF. (C, D) TUNEL staining (green) and DAPI (blue) in vehicle and ENT-treated cells (C). Quantitative analysis (D) shows a significant increase in the percentage of TUNEL-positive cells following ENT treatment. (E, F) WB

confirming increased MYOF protein levels in cells transfected with the MYOF overexpression (OE) vector compared to the empty vector (EV) control. (G–I) MTT assay results for U251 (G), U87 (H), and LN229 (I) cell lines over 7 days. Overexpression (OE) of MYOF partially rescues the growth inhibition induced by ENT treatment. (J–O) Transwell assays for U251 (J, K), U87 (L, M), and LN229 (N, O). Representative images (K, M, O) and quantification (J, L, N) demonstrate that MYOF overexpression mitigates the suppressive effects of ENT on cell motility. (P, Q) WB of P-STAT3 (Tyr705) and P-STAT3 (Ser727) in the cytoplasm and nucleus. GAPDH and Lamin B1 were used as cytoplasmic and nuclear loading controls, respectively. (R) Subcellular localization of P-STAT3 (green) and DAPI (blue) across different treatment groups. EV: Empty Vector. The data are presented as mean ± SD. *P<0.05, **P<0.01, ***P<0.001; one-way ANOVA, three independent experiments. Scale: 100μm.

Supplementary Table 1. shRNA synthesis sequence：

shRNA	Sequence (5’ to 3’)
shMYOF#1	TGCTGTTGACAGTGAGCGACCGGCGGATGCTGTCAAATAATAGTGAAGCCACAG ATGTATTATTTGACAGCATCCGCCGGCTGCCTACTGCCTCGGA
shMYOF#2	TGCTGTTGACAGTGAGCGCCAGGTTCTAAAGGTTATATGATAGTGAAGCCACAGA TGTATCATATAACCTTTAGAACCTGATGCCTACTGCCTCGGA

Supplementary Table 2. Primer sequences used for qRT-PCR and sh-RNA amplify.

Gene symbol	Forward primer sequence（5'to3'）	Reverse primer sequence（5'to3'）
GAPDH	TGACATCAAGAAGGTGGTGAAGCAG	GTGTCGCTGTTGAAGTCAGAGGAG
MYOF	GGGGCTGCATCATATACAGTAAC	GCTCATCATAGGGGTCTGGG

Supplementary Table 3. CRISPR/Cas9 oligo sequence：

oligo	Sequence (5’ to 3’)
MYOF-1-oligo1	CACCGACCACACTCATAGACGGCGA
MYOF-1-oligo2	AAACTCGCCGTCTATGAGTGTGGTC
MYOF-2-oligo1	CACCGATAATTGTTTGGTCCCACGT
MYOF-2-oligo2	AAACACGTGGGACCAAACAATTATC
MYOF-3-oligo1	CACCGGGAGTACAAGACCTGATGTG
MYOF-3-oligo2	AAACCACATCAGGTCTTGTACTCCC
MYOF-4-oligo1	CACCGTGATGCCAAAGGCTTACAGG
MYOF-4-oligo2	AAACCCTGTAAGCCTTTGGCATCAC
